# Influence of Material Deprivation on Clinical Outcomes Among People Living with HIV in High-Income Countries: A Systematic Review and Meta-analysis

**DOI:** 10.1007/s10461-021-03551-y

**Published:** 2021-12-11

**Authors:** Vasiliki Papageorgiou, Bethan Davies, Emily Cooper, Ariana Singer, Helen Ward

**Affiliations:** 1 Patient Experience Research Centre, School of Public Health, Imperial College London, London, UK; 2 Department of Epidemiology and Biostatistics, School of Public Health, Imperial College London, London, UK; 3 School of Public Health, Imperial College London, London, UK

**Keywords:** Antiretroviral therapy, HIV, Meta-analysis, Social determinants of health, Socioeconomic factors, Systematic review, Viral suppression

## Abstract

Despite developments in HIV treatment and care, disparities persist with some not fully benefiting from improvements in the HIV care continuum. We conducted a systematic review to explore associations between social determinants and HIV treatment outcomes (viral suppression and treatment adherence) in high-income countries. A random effects meta-analysis was performed where there were consistent measurements of exposures. We identified 83 observational studies eligible for inclusion. Social determinants linked to material deprivation were identified as education, employment, food security, housing, income, poverty/deprivation, socioeconomic status/position, and social class; however, their measurement and definition varied across studies. Our review suggests a social gradient of health persists in the HIV care continuum; people living with HIV who reported material deprivation were less likely to be virologically suppressed or adherent to antiretrovirals. Future research should use an ecosocial approach to explore these interactions across the lifecourse to help propose a causal pathway.

**Supplementary Information:**

The online version contains supplementary material available at 10.1007/s10461-021-03551-y.

## Resumen

A pesar de los avances en el tratamiento y la atención al VIH, ciertas desigualdades persisten en estas áreas. Como resultado, algunas personas que viven con el VIH no se benefician plenamente de las mejoras en la atención del VIH a todos los niveles. Realizamos una revisión sistemática para explorar las asociaciones entre los determinantes sociales de la salud y los resultados del tratamiento del VIH (supresión viral y tratamiento de adherencia) en países de ingresos altos. Completamos un metaanálisis siguiendo un modelo de efectos aleatorios e incluyendo medidas consistentes de exposiciones. Identificamos 83 estudios observacionales elegibles para inclusión. Los determinantes sociales vinculados a las circunstancias materiales (privación) se identificaron como educación, empleo, seguridad alimentaria, vivienda, ingresos, pobreza / privación, situación / posición socioeconómica y clase social; sin embargo, su medición y definición variaron entre los estudios. Nuestro estudio sugiere que un gradiente de salud social persiste en la atención sanitaria del VIH a todos los niveles; las personas que viven con el VIH que declaran privación material tenían menos probabilidades de alcanzar la supresión viral o de ser adherente a los antirretrovirales. Futuras investigaciones deberían utilizar un enfoque ecosocial para explorar estas interacciones a lo largo del curso de la vida para ayudar a proponer una vía causal.

## Introduction

The social determinants of health describe the conditions in which a person is “born, grows, lives, works and ages” and operate alongside social hierarchy, socioeconomic position, power differentials and the wider socioeconomic and political context [1]. Less privileged and marginalised individuals experience poorer health outcomes than the general population which can, in part, be attributed to current and historical struggles with structural racism, discrimination, recurring inequalities and social exclusion [1–4]. Recent evidence suggests that a decade of government austerity has led to widening health, social and economic inequalities in countries, such as England, that have resulted in stalling life expectancy, declining social mobility and increased food insecurity [5, 6].

Inequalities persist among people living with HIV globally despite advancements in life expectancy, treatment and care. Some groups are disproportionately affected including migrants, the homeless and sex workers [7–11]. These disparities are underpinned by social and structural factors including gender, sexual orientation, ethnicity, racism and socioeconomic position which influence an individual’s agency and power within a specific context [12, 13]. A literature review by Burch et al*.* [14] reported that people living with HIV who had poorer socioeconomic status (SES) were more likely to have poorer virological and immunological responses to antiretroviral therapy (ART). The authors defined SES according to material factors (e.g., education, neighbourhood socioeconomic position) and the health system (e.g., health insurance) itself [1, 14]. In countries without universal healthcare systems, such as the US, publicly funded systems of care exist to support individuals who are uninsured access healthcare; for instance, the Ryan White HIV/AIDS Program [15, 16]. However, the funding, accessibility and eligibility requirements of these services are influenced by structural determinants including the political systems, structures, policies and leadership in which they exist and operate.

For people living with HIV, adhering to prescribed antiretrovirals is essential to maintain virological suppression and to reduce the risk of drug resistance [17, 18]. Early research [19] estimated that HIV-1 viral load (VL) can be reduced by approximately 99% within two weeks of treatment initiation (using protease inhibitors and reverse transcriptase inhibitors). The level of ART adherence required to reach viral suppression is now considered to be regimen-dependent and could be as low as 75% for some [20]. Successful treatment and viral suppression are the second and third UNAIDS 90:90:90 targets; several high-income countries including the UK, Denmark and the Netherlands met these targets before the deadline of 2020 [21, 22]. Referral to, and retention in, HIV care services is therefore critical. However, this is dependent on early diagnosis which is not always achieved; in the UK in 2019, it was estimated that approximately 42% of people living with HIV were diagnosed late, defined by a CD4 count at diagnosis of < 350 cells/mm^3^ [23, 24]. The percentage of late diagnoses varied according to age, ethnicity and mode of transmission, with the highest proportion (52%) among heterosexual men [23, 24].

Social determinants exist within complex, intersectoral systems, can be highly correlated and are driven by the context in which they are created and manifest [1, 25]. We build on the definition of social determinants by the WHO Commission on Social Determinants of Health (CSDH) framework to include the “intermediary determinants” of living circumstances, working conditions and food availability [1]. We frame our findings in relation to Krieger’s ecosocial theory which aims to understand how “health inequities constitute biological expressions of injustice” across societal and ecosystem levels, pathways and power differentials [13, 26–28]. Ecosocial theory attempts to unpick the complexity of interactions across ecologies; for instance, the scale of phenomena (including measured and unmeasured factors), how these are organised (hierarchies) and spatiotemporal dynamics which means they are restricted by the extent to which these have been previously theorised, conceptualised, inferred and explored [27]. Much like ecosocial theory, the social production of disease and/or political economy of health as well as psychosocial theory can help elucidate how and why diseases are unevenly distributed across societies as well as implications for action [27]. We focus on factors of material deprivation, which could be targeted by social and public health policies.

Our aim is to synthesise the evidence and identify the social determinants that have an impact on HIV treatment outcomes (specifically viral suppression, ART adherence) among people living with HIV in high-income countries.

## Methods

We conducted a systematic review using the PRISMA 2020 checklist (Additional file 1); a full protocol is published on PROSPERO (identification number: CRD42020171850) [29, 30]. We adapted the approach of Burch et al.[14] to provide a more recent examination of the association between social determinants and HIV treatment outcomes; however, we focus on observational studies (cohorts and cross-sectional studies), rather than randomised controlled trials, as these replicate real-world settings.

### Search Strategy and Selection Criteria

We searched MEDLINE, EMBASE, Global Health, HMIC, Cochrane Library, CINAHL, Web of Science, ProQuest and Scopus databases from date of creation (or first stored record) to 13 January 2020 using a search strategy developed with a University librarian. We also hand searched conference databases until March 2020 and searched the reference lists of relevant review articles and editorials. Further detail, including search strategies, are provided in Additional file 2.

Studies were assessed for eligibility using the criteria detailed in Additional file 3. To be included, study populations had to comprise adults (aged 18 or older) living with HIV in high-income countries. We defined high-income countries using the 2019 World Bank classification and Organisation for Economic Co-operation and Development (OECD) country membership [31, 32]. Social determinants focussed on measures of material deprivation and were broadly defined as education, employment, food security, housing, income, poverty (or deprivation), socioeconomic status (or position) and social class. They had to be compared across levels and recorded at either the individual, household, or neighbourhood level. The primary outcomes of interest were HIV treatment-related, specifically medication adherence and viral suppression, measured by VL or CD4 cell counts. We also extracted data of other social factors which may act as confounders, specifically age, gender, sexual orientation, ethnicity and migration status.

### Screening, Data Collection and Analysis

Two authors (VP, AS) screened title and abstracts, followed by full-text, using Covidence [33] and original study authors were contacted by VP to provide any unavailable full-text articles. If the search identified a non-peer-reviewed and peer-reviewed publication for the same study, the most recently published was included. Selected studies were then exported and managed using Excel with a data extraction table initially piloted among 10 studies and subsequently refined. VP extracted data items (Additional file 4) which were cross-checked by EC.

Forest plots were created using Revman 5 [34] for each social determinant and used to present relative effect sizes of comparable associations [adjusted odds ratios (aOR) with 95% confidence intervals (CIs)]. Data are mainly presented using a narrative synthesis as there were large amounts of heterogeneity between included studies. A random-effects meta-analysis was performed for studies where definitions and measurements across studies were consistent; we present the associated I^2^ value for heterogeneity (proportion of variation in effect estimates due to heterogeneity rather than chance) [35]. Data presented in forest plots compare poorer social determinants (e.g., unstable housing) to improved determinants (e.g., stable housing).

### Quality Assessment

VP and BD assess the risk of bias of included studies using the Newcastle–Ottawa scale and an adapted version for cross-sectional studies (Additional file 5) [36, 37]. Studies categorised as ‘other’ study design, such as secondary data analysis, are assessed using the most appropriate quality assessment tool. We define the most important confounders for studies to adjust for as gender, sexual orientation, age, race/ethnicity and social class, based on the literature [38]. We define adequate follow-up for outcomes based on WHO guidelines of routine VL monitoring of 6 months following ART initiation and measuring ART adherence at 30 days [39, 40]. No data from ‘low quality’ studies are included in the meta-analysis; therefore, a sensitivity analysis was not conducted.

## Results

**Fig. 1:**
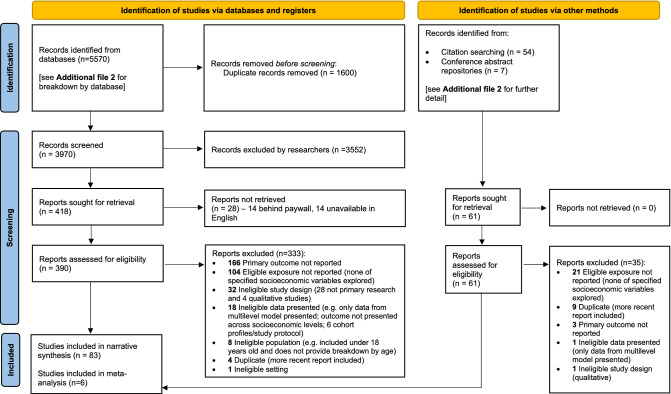
PRISMA 2020 flow diagram. Adapted from Page et al. [29]

We screened 4031 records, following the removal of duplicates, of which 83 observational studies were eligible (Fig. 1).

Most included studies explored the social determinants of education (n = 52, 62.7%); followed by housing (n = 39, 47.0%), employment (n = 33, 39.8%) and income (n = 33, 39.8%) (Table 1). The studies included a total of 1,445,150 people living with HIV. Almost three-quarters (n = 61, 73.5%) of the included studies were based in North America. Some studies focussed on specific sub-populations of people living with HIV such as transgender women (n = 5, 6.0%), people who formerly/currently use drugs (n = 3, 3.6%), people in prison (n = 1, 1.2%), migrants (n = 1, 1.2%), people living with HIV and hepatitis C (n = 1, 1.2%), homeless/marginally housed individuals (n = 1, 1.2%) as well as individuals who hold multiple identities; for instance, being in prison and homeless (n = 1, 1.2%) or being socioeconomically disadvantaged and using drugs (n = 1, 1.2%).

**Table 1:** Summary of key characteristics of included studies

Characteristic	Total studies (%)
Publication type	
Peer-reviewed (e.g., journal article, short/brief report, short/concise communication)	77 (92.8)
Not peer-reviewed (e.g., conference abstract, editorial letter, thesis)	6 (7.2)
Setting	
North America	61 (73.5)
Europe	20 (24.1)
Asia	1 (1.2)
Australia	1 (1.2)
Study design	
Cohort/longitudinal	31 (37.3)
Cross-sectional^b^	30 (36.1)
Other^c^	22 (26.5)
Social determinants^a^	
Education	52 (62.7)
Employment	33 (39.8)
Food security	7 (8.4)
Housing	39 (47.0)
Income	33 (39.8)
Poverty/deprivation	9 (10.8)
Socioeconomic status/position	4 (4.8)
Social class	1 (1.2)
Primary outcome(s)	
Viral (non-)suppression^d^	38 (45.8)
Medication (non-)adherence^e^	35 (42.2)
Both	10 (12.0)
Secondary outcome(s)^a^	
Diagnosis-related	1 (1.2)
Medication-related (e.g., initiation, use, coverage, response)	8 (9.6)
HIV care-related (e.g., engagement, retention, missed visits)	13 (15.7)

^a^More than 1 option possible

^b^Includes cross-sectional surveys of cohort studies

^c^Mixed methods (observational data extracted), programme evaluations, needs assessment, cross-sectional surveys/analysis of cohort studies and chart/record/baseline intervention reviews

^d^CD4 cell count, HIV viral load

^e^ART, cART, HAART

We identified 38 studies that explored the association between social determinants and virological suppression (or non-suppression); 35 that investigated adherence (or non-adherence) to ART including combination ART (cART) and highly active ART (HAART); and 10 that explored both primary outcomes. Study characteristics are detailed in Table 2.

**Table 2:** Characteristics of included studies

	Population		Exposure	Outcomes	
Study (publication type)	Setting (city/state, country)	Population of people living with HIV; project name (study design)	Social determinant(s)^a^	Virological (non-) suppression measure^b^	Medication^c^ (non-) adherence measure
North America (n = 61)					
Almeida-Brasil et al. 2018 [42] (article)	Canada	566 adults with hepatitis C, receiving cART; part of Food Security & HIV-HCV Study (FS) of the Canadian Co-infection Cohort (CCC) (prospective cohort)	Education; employment; food security; income	VL > 50 copies/mL	Missed ≥ 1 cART dose, past 4 days
Anderson et al. 2018 [43] (article)	USA	239 women at an urban HIV specialty clinic (cross-sectional)	Education; employment	CD4 < 200 cells/mm^3^ VL > 20 copies/mL	–
Baguso et al. 2019 [44] (article)	San Francisco (USA)	123 self-identified transgender women; part of Transwomen Empowered to Advance Community Health 3 (TEACH 3) study (cross-sectional)	Education; housing	Detectable and unknown viral load	–
Berg et al. 2004 [45] (article)	Bronx, New York (USA)	113 people who currently/formerly use opioids; part of HIV Epidemiologic Research on Outcomes (HERO) study (prospective cohort)	Housing	–	MEMS caps openings divided by number of prescribed doses
Blank et al. 2015 [46] (article)	Brooklyn; Chicago; Los Angeles; Miami; San Antonio; Longview; Anniston; and Chapel Hill (USA)	944 women of colour; part of Special Projects of National Significance (SPNS) Enhancing Access to and Retention in Quality HIV Care for Women of Color initiative (prospective cohort)	Education; employment; housing	VL < 200 copies/mL	–
Chitsaz et al. 2013 [47] (article)	Connecticut; Georgia; Illinois; Massachusetts; New York; Ohio; Pennsylvania; Rhode Island; South Carolina (USA)	1166 adults with a diagnosis of HIV before being jailed in 10 jails in US across 9 states; part of Enhance Link initiative (cross-sectional)	Education; employment; food security; housing	–	≥ 95% ART adherence
Clemenzi-Allen et al. 2018 [48] (brief report)	San Francisco, USA	1222 adults at HIV clinic (“Ward 86”) (cross-sectional)	Housing	VL < 200 copies/mL	–
Creasy et al. 2019 [49] (article)	Atlanta; Detroit; Houston; Memphis; Philadelphia; and Washington, DC (USA)	5143 male and Black/African American adults recruited from Black Pride events; part of Promoting Our Worth, Equality, and Resilience (POWER) study (cross-sectional)	Housing	–	Missed doses (4–7/week, 2–3/week, 1/week, < 1/week, never) Last missed dose (never, ˃ 3 months,1–3 months, 3–4 weeks, 1–2 weeks, or within the week)
Doshi et al. 2017 [50] (article)	USA	1,296,248 adults accessing ˃800 medical care providers, 2010–2014; part of Health Resources and Services Administration (HRSA) and served by Ryan White program (surveillance records review)	Housing	VL < 200 copies/mL	–
Dowshen et al. 2016 [51] (short report)	USA (including Puerto Rico), 15 cities	1584 “behaviourally-infected youth” (including 66 young transgender women) from a multisite study conducted at 20 Adolescent Medicine Trials Units; part of Adolescent Trials Network (ATN) (secondary data analysis—cross-sectional)	Housing	Detectable viral load	–
Fadul et al. 2017 [52] (conference abstract)	North Carolina, USA	184 newly diagnosed people living with HIV treated in East Carolina University clinic; part of Ryan White program (retrospective chart review)	Education; housing; poverty	VL < 200 copies/mL	-
Feldman et al. 2015 [53] (article)	Greater New York metropolitan area (USA)	2896 MSM; part of Ryan White Part A program (programme evaluation)	Education; housing	VL > 200 copies/mL CD4 < 350 cells/mm^3^	–
Feller and Agins 2016 [54] (article)	New York State (USA)	11,252 adults receiving treatment across 186 HIV clinics (classification and regression tree analysis algorithm—cohort)	Housing	VL < 200 copies/mL % virally suppressed	–
Gardner et al. 2015 [55] (conference abstract)	Ontario (Canada)	3322 people living with HIV receiving clinical care; part of Ontario HIV Treatment Network Cohort Study (OCS) (cohort)	Income	VL < 200 copies/mL	–
Gebo et al. 2003 [56] (article)	USA	196 people living with HIV taking at least 1 ART in an urban hospital clinic (John Hopkins University HIV Clinic) (cross-sectional)	Deprivation	–	< 90% ART adherence (stratified by injecting drug use and gender)
Golin et al. 2002 [57] (article)	Southern California (USA)	117 people living with HIV in a county hospital HIV clinic with newly initiated HAART; part of Adherence and Efficacy to Protease inhibitor Therapy (ADEPT) study (prospective cohort)	Education; employment; income	–	Number of PI/NNRTI/HAART doses taken divided by prescribed over 4-weeks
Haider et al. 2019 [58] (article)	South Carolina (USA)	342 people living with HIV receiving HIV care in immunology centre; part of Ryan White Program (cross-sectional)	Education; employment; income	VL < 100 copies/mL	–
Hussen et al. 2018 [59] (article)	Southeastern city, USA	81 young Black gay, bisexual and other MSM aged 18–24 years old in a paediatric/adolescent clinic (cross-sectional)	Education; employment; housing	VL ≤ 40 copies/mL	–
Iralu et al. 2010 [60] (article)	Navajo Nation (USA)	36 American Indians under Navajo AIDS Network (NAN) case management; part of Four Corners American Indian Circle of Services Collaborative (4CC) (cross-sectional)	Education; employment; housing; income	CD4 < 200 cells/mm^3^ Viral load (log)	–
Johnson et al. 2003 [61] (article)	San Francisco; Los Angeles; New York City; Milwaukee, (USA)	2765 adults taking ART; part of Healthy Living Project (baseline pre-intervention—cross-sectional)	Education; employment; housing	–	90% ART adherence, past 3 days
Kacanek et al. 2019 [62] (article)	USA (including Puerto Rico)	122 18–22-year olds based across 15 clinical sites; part of Pediatric HIV/AIDS Cohort Study (longitudinal)	Income	VL > 400 copies/mL	Self-reported missed ≥ 1 ART dose, past week
Kalichman and Grebler 2010 [63] (article)	Atlanta, Georgia (USA)	188 people living with HIV/AIDS who demonstrated poor health literacy (cross-sectional)	Education; employment; income; poverty	–	85% ART adherence 75% ART adherence
Kalichman et al. 2010 [64] (article)	Atlanta, Georgia (USA)	344 people living with HIV/AIDS (cross-sectional)	Education; employment; food security; housing; income	Undetectable viral load Change in most recent viral load Change in most recent T cells Most recent CD4 cell count	80% ART adherence 90% ART adherence Self-report adherence rating Unannounced pill count adherence
Kalichman et al. 2014 [65] (article)	Atlanta, Georgia (USA)	364 men and 157 women living with HIV (cross-sectional)	Food security	CD4 < 500 Detectable viral load	85% ART adherence
Keith McInnes et al. 2013 [41] (article)	Atlanta; Baltimore; Bronx; Brooklyn/Manhattan; Houston; Los Angeles; Pittsburgh; and Washington, DC (USA)	1871 veterans living with HIV; part of Veterans Aging Cohort Study (VACS) and completed 5th follow-up survey in 2010–2011 (cross-sectional analysis of longitudinal cohort)	Education; employment; housing; income	–	ART adherence (1-MEDOUT ≥ 0.90)
Kleeberger et al. 2004 [66] (concise communication)	USA	597 men living with HIV reporting use of HAART; part of Multicenter AIDS Cohort Study (prospective cohort)	Education; income	–	Negative change (%): visit-pairs with 100% adherence Positive change (%): visit-pairs with < 100% adherence
Koehn et al. 2020 [67] (short communication)	Vancouver (Canada)	99 Dr Peter Centre clients who completed a 12-month follow-up interview (longitudinal cohort)	Education; employment; food security; housing; income	–	≥ 95% cART adherence
Kyser et al. 2011 [68] (article)	Denver; Minneapolis; Providence; and St. Louis (USA)	528 people living with HIV taking cART; part of study to understand the natural history of HIV/AIDS in the era of effective therapy (SUN) (cross-sectional analysis of longitudinal, prospective cohort)	Education; employment	–	cART adherence, past 3 days (missed ≥ 1 ART doses)
Lacombe-Duncan et al. 2019 [69] (article)	British Columbia, Ontario, and Quebec (Canada)	50 transgender women; part of Canadian HIV Women’s Sexual and Reproductive Health Cohort Study (CHIWOS) (cross-sectional / mixed methods)	Education; housing; income	VL < 50 copies/mL	≥ 95% ART adherence
Lim et al. 2015 [70] (article)	New York City (USA)	1698 people living with HIV /AIDS with both jail incarceration and homelessness (retrospective cohort)	Housing	VL < 400 copies/mL	–
Ludema et al. 2016 [71] (article)	Bronx; Brooklyn; Washington DC; Los Angeles; San Francisco; Chicago (USA)	1481 women; part of Women’s Interagency HIV Study (WIHS) (prospective cohort)	Income	VL > 200 copies/mL	–
Marshall et al. 2016 [72] (article)	Vancouver (Canada)	706 people who use drugs; part of AIDS Care Cohort to Evaluate Exposure to Survival Services (ACCESS) (prospective cohort)	Education; employment; housing	Undetectable VL or VL < 50 copies/mL	–
McCoy et al. 2016 [73] (article)	Arizona, California, Illinois, Massachusetts, Michigan, Pennsylvania, Texas, Washington, and Wisconsin (USA)	426 people living with HIV aged 50 years and older; part of PRIME baseline study (baseline pre-intervention—cross-sectional)	Education; employment; income	–	≥ 95% ART adherence
Miller et al. 2006 [74] (article)	British Columbia (Canada)	892 people living with HIV; part of HIV/AIDS Drug Treatment Program of the British Columbia Centre for Excellence in HIV/AIDS (programme evaluation)	Housing; income	VL < 500 copies/mL	–
Mimiaga et al. 2019 [75] (article)	Rhode Island (USA)	296 adults; part of Ryan White Part B HRSA (needs assessment)	Education; employment; housing; income	Virological non-suppression	Sometimes/never take ART as prescribed, past 12 months
Mohammed et al. 2004 [76] (article)	Louisiana (USA)	273 adults from 8 areas in non-urban Louisiana (cross-sectional)	Education; employment	–	Self-report missed any HAART doses, previous week
Moore et al. 2016 [77] (article)	Vancouver (Canada)	719 MSM; part of Momentum Health study (cross-sectional)	Education; income	VL ≥ 200 copies/mL	–
Nyaku, Beer and Shu 2019 [78] (article)	USA (including Puerto Rico)	18,423 adults who self-reported taking ART: part of Medical Monitoring Project (MMP) (secondary data analysis—surveillance records review from cross-sectional sample)	Education; housing; poverty	–	ART non-persistence
O'Neil et al. 2012 [79] (article)	British Columbia (Canada)	566 people living with HIV who have accessed ART; part of Longitudinal Investigations into Supportive and Ancillary health services (LISA) cohort study (cross-sectional analysis of cohort)	Education; housing; income	–	≥ 95% HAART adherence
Oliver et al. 2019 [80] (article)	Nashville, Tennessee (USA)	248 women living with HIV with ≥ 1 prenatal visit; part of Vanderbilt Obstetrics Comprehensive Care Clinic (VCCC) (observational cohort)	Education	VL ≥ 200 copies/mL	–
Phillips 2011 [81] (article)	USA	160 Black men living with HIV/AIDS who use illicit drugs (cross-sectional)	Housing	–	Self-reported mean number of ART doses missed, past 4 days
Phillips et al. 2013 [82] (article)	Canada; USA (including Puerto Rico)	1873 adults; part of International Nursing Network for HIV Research Study V (cross-sectional)	Education	–	≥ 99% ART adherence (self-reported visual analogue scale for 30 days)
Rebeiro et al. 2018 [83] (article)	Southern USA	2541 adults in viral suppression analysis; part of VCCC (observational cohort)	Socioeconomic status/position	VL < 200 copies/mL among those with ≥ 1 clinic visit	–
Robinson and Knowlton 2016 [84] (article)	Baltimore (USA)	383 people who inject drugs formerly/currently (secondary data analysis—cross-sectional)	Education	VL ≤ 40 copies/mL	-
Santos et al. 2014 [85] (short report)	San Francisco (USA)	314 transgender women; part of San Francisco Transfemale Respondent Driven Sampling Study (secondary data analysis—cross-sectional)	Housing	VL ≤ 200 copies/mL	–
Sayles et al. 2012 [86] (article)	Los Angeles county (USA)	11,397 people living with HIV who were uninsured; part of Ryan White program (cohort)	Housing; income	VL > 200 copies/mL	–
Schafer et al. 2012 [87] (article)	Virginia (USA)	251 men and women; part of UVa Ryan White clinic (RWC) (cross-sectional cohort)	Education; socioeconomic status	CD4 < 200 Detectable HIV viral load	–
Shacham et al. 2010 [88] (article)	St Louis, Missouri (USA)	514 people living with HIV presenting at Washington University HIV Clinic in 2007 (cross-sectional)	Education; employment; housing	VL ≥ 400 copies/mL	–
Shacham et al. 2013 [89] (article)	St Louis, Missouri (USA)	762 people living with HIV presenting at Washington University HIV Clinic in 2008 (cross-sectional)	Employment; poverty	CD4 < 200 cells/μl VL < 400 copies/mL	–
Singh et al. 1999 [90] (article)	USA	123 people who completed refill-methodology assessment of adherence (prospective cohort)	Education; employment; income	–	< 90% ART adherence
Stone et al. 2001 [91] (article)	Rhode Island; Maryland; New York State; Michigan (USA)	289 women living with HIV/AIDS; part of HIV Epidemiologic Research (HER) longitudinal cohort study (cross-sectional survey of cohort)	Education	–	Missed ≥ 1 HAART doses, past 3 days
Storholm et al. 2019 [92] (article)	Los Angeles, California (USA)	239 African American adults living with HIV recruited in community settings (246: Project Mednet and 108 from Project Rise) (longitudinal)	Education; employment; housing; income	–	ART adherence trajectory group: high-stable (mean > 90%), moderately low-stable (> 60%), low-decreasing (< 27%) at 2, 4, 6 months or 1.5, 4.5, 6 months
Sunil and McGehee 2007 [93] (article)	USA	1910 people living with HIV taking ART; part of HIV Cost and Services Utilization Study (HCSUS) (cross-sectional survey of cohort)	Education	–	ART adherence, past 7 days (non-adherent: ≥ 1 medication missed)
Surratt et al. 2015 [94] (article)	Miami; Fort Lauderdale (USA)	503 socioeconomically disadvantaged substance users (cross- sectional—structured interviews)	Housing; income; poverty	–	Missed ARTs because of diversion, past 90 days
Tymejczyk et al. 2018 [95] (article)	New York City (USA)	1045 people living with HIV (sexual health clinic electronic medical records matched with longitudinal data from NYC HIV Surveillance Registry in 12 months before and after clinic visits) (secondary data analysis—linked surveillance and medical records review)	Poverty	VL ≤ 200 copies/mL	–
Vyas et al. 2014 [96] (article)	San Diego, California (USA)	350 adults attending a HIV clinic (prospective longitudinal)	Income	–	≥ 90% ART adherence
Wagoner et al. 2016 [97] (article)	Birmingham, Alabama (USA)	382 people living with HIV at University of Alabama clinic (1917 Clinic) with a HIV viral load available; part of Project Client-Oriented New Patient Navigation to Encourage Connection to Treatment (CONNECT) (cohort)	Education	VL ≥ 200 copies/mL	–
Weiser et al. 2013 [98] (concise communication)	San Francisco (USA)	284 homeless and marginally housed individuals; part of San Francisco Research on Access to Care in the Homeless cohort (observational cohort)	Education; food security; housing; income	VL > 100 copies/mL CD4 < 200 cells/mm^3^	< 90% ART adherence
Whelan et al. 2019 [99] (article)	King County, Washington (USA)	549 newly diagnosed people living with HIV who received a partner services interview (retrospective cohort)	Housing	VL ≥ 200 copies/mL	–
Wilson et al. 2018 [100] (article)	San Francisco Bay Area (USA)	159 transgender women of colour (55 participated at TransAccess, 46 at Brandy Martell Project, and 58 at Butterfly Project) as part of the Special Projects of National Significance (SPNS) Program, “Enhancing Engagement in Retention and Quality of Care for Transgender Women of Color” initiative (programme evaluation)	Food security; housing; income	CD4 < 500, past 6 months Undetectable VL, past 6 months	–
Yehia et al. 2014 [101] (article)	Philadelphia, Pennsylvania (USA)	12,759 adults using multiple clinics for primary HIV care; part of Ryan White program (retrospective cohort)	Income	VL ≤ 200 copies/mL	–
Europe (n = 20)					
Abgrall et al. 2014 [102] (letter)	France	200 individuals from Sub-Saharan Africa adherent to cART at enrollment; part of Agence Nationale de Recherche sur le SIDA et les hépatites virales (ANRS)-VIHVO study (cross-sectional)	Housing	–	< 80% cART adherence
Burch 2018 [103] (thesis)	UK	2704 adults recruited from eight HIV outpatient clinics; part of Antiretrovirals Sexual Transmission Risk and Attitudes (ASTRA) study (cross-sectional)	Education; employment; housing; income	VL > 50 copies/mL VL > 200 copies/mL	≥ 2 ART consecutive missed days, past 3 months or ≥ 1 missed, past 2 weeks
Carrieri et al. 2003 [104] (article)	Marseilles; Avignon; Nice; Paris suburbs (France)	96 people who inject drugs (30 women, 66 men) living with HIV initially adherent to HAART in hospital clinics; part of French MANIF 2000 cohort (cohort)	Education; employment; housing	–	< 80% HAART adherence, past 18-months
Collazos et al. 2009 [105] (article)	Spain	1352 people living with HIV from 69 hospitals; part of Grupo Español para el Estudio Multifactorial de la Adherencia (GEEMA) cohort (prospective cohort)	Education	CD4 + cell count (cells/µl) at baseline Undetectable VL (baseline and 12 months) VL (log copies/mL) at baseline	HAART adherence rates
D'Almeida et al. 2016 [106] (article)	France	1246 people living with HIV who were HIV treatment-naïve at cART initiation and on cART for at least 12 months across 73 hospital departments; part of ANRS VESPA-2 study (cross-sectional)	Education; employment; deprivation	Sustained viral suppression (or undetectable VL < 50 copies/mL) for at least 6 months	–
Del Amo et al. 2017 [107] (article)	Austria, France, Germany, Greece, Italy, Spain, Switzerland, and the Netherlands	24,069 people living with HIV with data on education from 15 cohorts of patients initiating cART; part of Collaboration of Observational HIV Epidemiological Europe (COHERE) within EuroCoord Network of Excellence (multi-site cohort)	Education	VL < 400 copies/mL (1 year after cART initiation) Immunological response (change in CD4 + count) at baseline, first 6 months and after 6 months	–
Dorz et al. 2003 [108] (article)	Italy	88 male and 21 female adults living with HIV undergoing protease inhibitor treatment (cross-sectional)	Education; employment; income	–	< 80% adherence, past 7 days
Gordillo et al. 1999 [109] (article)	Madrid (Spain)	366 people living with HIV on ART treatment (cross-sectional)	Education; employment	–	> 90% ART adherence ('good’)
Gueler et al. 2015 [110] (article)	Switzerland	2694 ART-naïve for virologic response at six months; part of Swiss HIV Cohort Study (prospective cohort)	Socioeconomic position	Virological response to cART or viral suppression (VL < 50 copies/mL) at 6 months	–
Jansen et al. 2009 [111] (conference abstract)	Germany	2045 people living with HIV; part of Competence Network for HIV/AIDS (KompNet) cohort (cohort)	Income	VL < detection limit CD4 + cell count	–
Papadopoulou 2000 [112] (thesis)	Enfield/Haringey, London (England)	56 people living with HIV taking cART (cross-sectional)	Education	–	cART adherence (4 categories—“adhering to correct dose”, “taking drugs at right time”, “following dietary instructions” and “overall adherence”)
Parruti et al. 2006 [113] (article)	Abruzzo Region (Italy)	171 people living with HIV clinically monitored for at least 24 weeks (cohort)	Employment; housing; SES	–	90% HAART adherence
Persson et al. 1994 [114] (article)	Malmö (Sweden)	47 MSM living with HIV (cross-sectional)	Social class	Low CD4 cell count (< median)	–
Raho-Moussa et al. 2019 [115] (article)	Paris (France)	475 people living with HIV attending two hospital outpatient clinics on ART for at least 6 months (cross-sectional)	Education; employment; deprivation	VL > 50 copies/mL	–
Saracino et al. 2018 [116] (article)	Italy	8023 ART naïve people living with HIV with Italian nationality; part of Italian Cohort Naïve Antiretrovirals (ICONA) cohort (observational cohort)	Education; employment	VL < 50 copies/mL	ART discontinuation
Sellier et al. 2006 [117] (brief report)	Paris (France)	61 adults born in Sub-Saharan Africa and living in France attending 3 infectious diseases departments (cross-sectional)	Education; employment	–	Never missed ART doses compared to frequently or rarely
Sherr et al. 2012 [118] (article)	London (UK); South East (UK)	259 adults living with HIV attending 5 HIV clinics (cross-sectional)	Education	–	All HAART doses taken at correct time and under correct conditions
Sobrino-Vegas et al. 2012 [119] (article)	Spain	4549 people living with HIV from 27 public health centres; part of CoRIS (AIDS Research Network Cohort) (prospective cohort)	Education	Immunological response to treatment (CD4 + T-cell count) at 6 months and 1 year VL < 50 copies/mL (6 months and 1 year)	–
Spire et al. 2002 [120] (article)	France	445 people living with HIV in 47 hospital departments; part of APROCO cohort (prospective cohort)	Housing; income	–	HAART non-adherence at 4 months follow up
Uusküla et al. 2012 [121] (article)	Kohtla-Järve (Estonia)	144 people living with HIV receiving outpatient HIV care (cross-sectional)	Education; income	–	< 100% ART adherence
Asia and Australia (n = 2)					
Siefried et al. 2017 [122] (article)	Australia	522 adults on ART at 7 clinics; part of Parameters Associated with Adherence to Antiretroviral Therapy (PAART) study (cross-sectional analysis of cohort)	Employment; housing; income	–	Self-reported missed ≥ 3 ART, past 3 months
Yang and Bang 2017 [123] (article)	South Korea	300 people living with HIV from 6 hospitals; part of Nationwide Specialized Counseling Program (cross-sectional)	Education; employment; income	–	≥ 95% ART adherence

*ART* antiretroviral therapy, *cART* combination antiretroviral therapy, *HAART* highly active antiretroviral therapy, *MEMS* medication event monitoring system, *MSM* men who have sex with men, *PI* protease inhibitor, *VL* viral load

^a^Measures of social determinants vary across studies and may be compared at individual, household, or neighbourhood level

^b^Measures of viral suppression include CD4 cell count and viral load. ^c^Medication includes ART, cART, and HAART

Most studies measuring viral suppression predominantly recorded VL, although some also reported the immunological response of CD4 cell count. The VL thresholds to meet viral suppression varied across studies from 20 to 400 VL copies/mL or defined as ‘detectable’ or ‘undetectable’. Adherence had more varied measurements; some studies asked how often participants had missed medication in a specific time frame or used medication event monitoring systems (MEMS) to record the number of times a pill cap was opened which was sometimes also verified by an unannounced pill count by phone. One study [41] used an index of the “number of days out of medication” (MED-OUT) using pharmacy-refill based measures.

Social determinants primarily focussed on measures of material deprivation. This included education (e.g., level, attainment, student status); employment (e.g., status, grade, type); housing (e.g., status, stability, homelessness, living situation, condition, ownership); measures of food security; income (e.g., annual household, financial stability, receiving benefits, financial hardship/concerns, economic situation); socioeconomic status/position (individual/neighbourhood); and measures of deprivation and social class.

We present data under the following headings: education, employment, housing, and material deprivation (which includes measures of deprivation, food security, income, and socioeconomic status/position). For some studies, we present the inverse of the data reported in the original manuscript as we were interested in the outcomes of virological suppression and ART adherence. A full summary of findings, including the confounders adjusted for and reference categories, is provided in Additional file 6.

### Education

Education was typically defined by the highest educational level of attainment; from primary school in the UK (or elementary school in the US) to university qualifications.

**Fig. 2:**
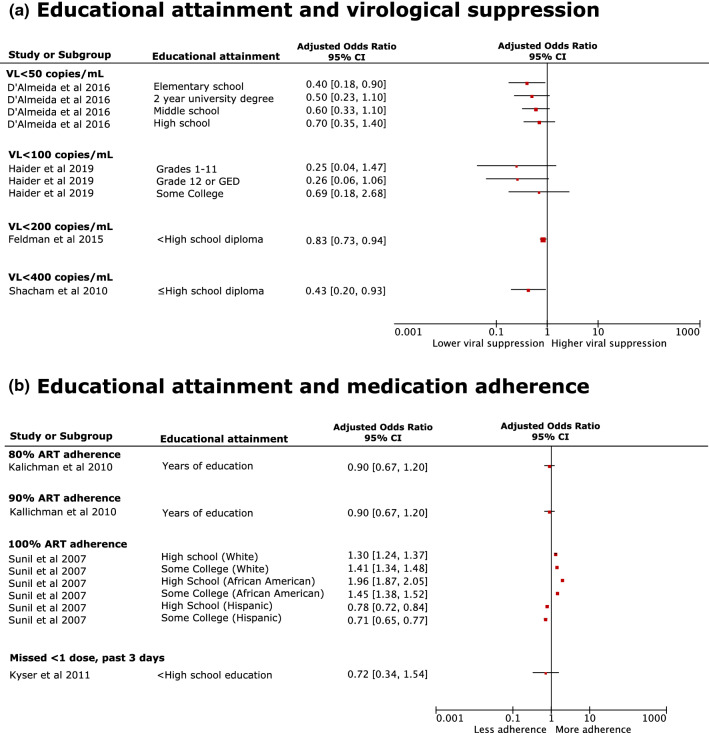
Forest plots of likelihood (aOR) of **a** virological suppression and **b** medication adherence among people living with HIV with low, compared to high, educational attainment after adjusting for sociodemographic factors. *ART* antiretroviral therapy, *GED* general educational diploma, *VL* viral load

Of 83 studies, 52 focussed on education with nine studies reporting a significant adjusted association with virological suppression [43, 84, 88, 97, 103, 106, 107, 116, 119]; four reported negative associations (lower education) and five positive (higher education). Overall, people who had lower educational levels were less likely to be virologically suppressed (Fig. 2a; Additional file 6) than individuals who had higher educational attainment after adjusting for confounders [53, 88, 106]. For instance, in a cohort of 1246 people living with HIV attending healthcare in France, D’Almeida et al. [106] found individuals whose highest educational attainment was elementary school were 60% less likely (aOR 0.40; 95% CI 0.18, 0.90) to have a VL < 50 copies/mL, compared to those with more than 2 years of a university degree. Shacham et al.[88] found that individuals presenting at an urban HIV clinic in the US, with a high school diploma or less were over 2-times more likely (aOR 2.32; 95% CI 1.08, 5.00) to be virologically non-suppressed; this study used a higher threshold (VL < 400 copies/mL).

The direction of the association between education and medication adherence was less clear. Four studies reported a significant adjusted negative association with medication adherence and lower educational attainment [57, 93, 103, 118]; one [93] also reported positive associations among some sub-groups (Fig. 2b; Additional file 6). Sunil and McGehee [93] found different patterns across educational levels when stratifying by race/ethnicity. White Americans (n = 992) who had completed high school, some College or had an undergraduate qualification were 30% (aOR 1.30; 95% CI 1.24, 1.37), 41% (aOR 1.41; 95% CI 1.34, 1.48) and 58% (aOR 1.58; 95% CI 1.51, 1.66) more likely, respectively, to be adherent to ART compared to individuals who completed some high school only [93]. The same general trend was seen for African American participants (n = 581): individuals who completed high school and some College were 96% (aOR 1.96; 95% CI 1.87, 2.05) and 45% (aOR 1.45; 95% CI 1.38, 1.52) more likely to be adherent to ART than those who only completed some high school, however those with an undergraduate qualification were 12% less likely (aOR 0.88; 95% CI 0.82, 0.95) to be adherent [93]. For Hispanic American participants (n = 272), the direction of association was less sequential; individuals who had completed high school or some College were 22% (aOR 0.78; 95% CI 0.72, 0.84) and 29% (aOR 0.71; 95% CI 0.66, 0.77) less likely, respectively, to be adherent whereas university graduates were 40% more likely (aOR 1.40; 95% CI 1.27, 1.55) to be adherent compared to those who only completed some high school [93].

### Employment

Employment was defined either by status, occupation type or whether work was paid.

**Fig. 3:**
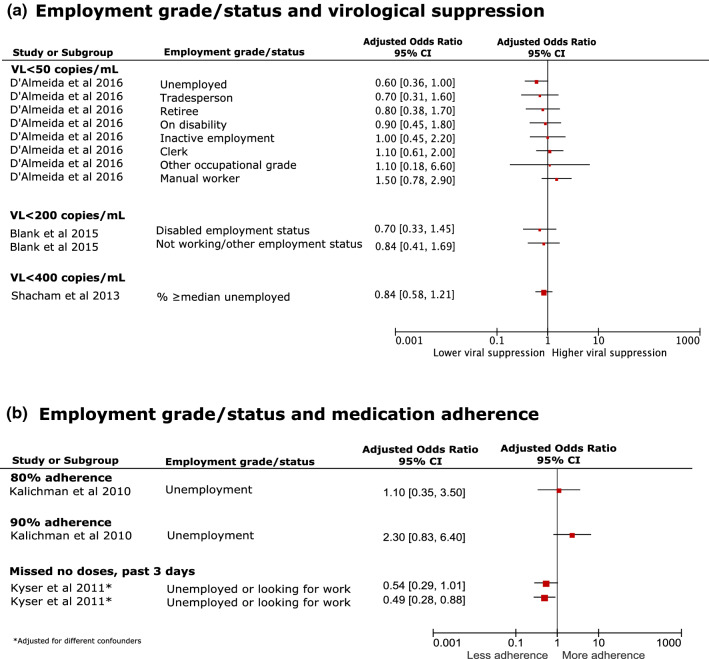
Forest plots of likelihood (aOR) of **a** virological suppression and **b** medication adherence among people living with HIV with lower employment grade or unemployed status, compared to high, after adjusting for sociodemographic factors. *VL* viral load

Of 83 studies, 33 looked at employment with only three studies reporting a significant association with virological suppression after adjustment for confounders [58, 103, 116]; two associations were negative (those unemployed), one was positive (those employed) (Fig. 3a; Additional file 6). Overall, there was an inconsistent association between employment and virological suppression. For instance, D'Almeida et al.[106] reported no difference in viral suppression between people living with HIV of a lower employment grade or unemployed working status compared with executive occupational grades or employed. However, Saracino et al. 2018 [116] found that unemployed people living with HIV had lower rates (adjusted Hazard Ratio [aHR] 0.87; 95% CI 0.79, 0.96) of virological suppression, compared to full-time workers. Similarly, Burch [103] reported that the prevalence of virological non-suppression (VL > 50 copies/mL) was almost 2-times greater among the unemployed, compared to employed (adjusted Prevalence Ratio [aPR] 1.98; 95% CI 1.51, 2.61).

Similarly, the association between medication adherence and employment varied. Four studies reported a significant adjusted association with medication adherence [47, 68, 103, 116]; three reported a negative association (those unemployed) and one a positive association (those employed) (Fig. 3b; Additional file 6). Saracino et al.[116] found unemployed people living with HIV were more at risk of discontinuing ART (aHR 1.18; 95% CI 1.04, 1.34) and when they looked at specific job types, “housewives” were found to be less at risk of ART discontinuation (aHR 0.73; 95% CI 0.59, 0.90), after adjusting for CD4 count, VL, pregnancy status and smoking. Kyser et al.[68] reported that people living with HIV who were unemployed or looking for work were over 2-times more likely (aOR 2.03; 95% CI 1.14, 3.61) to report having missed cART doses in the past 3 days.

### Housing

Some studies described housing as the physical environment; some grouped individuals into categories of ‘unstable’ and ‘stable’ housing; some used time of residence and others used home ownership. We grouped studies which explored the association between people living with HIV in unstable compared to stable housing and differing VL thresholds. Housing ‘stability’ was either explicitly described by the authors or was defined in relation to the type of housing (e.g., living in subsidised housing or in a shelter).

**Fig. 4:**
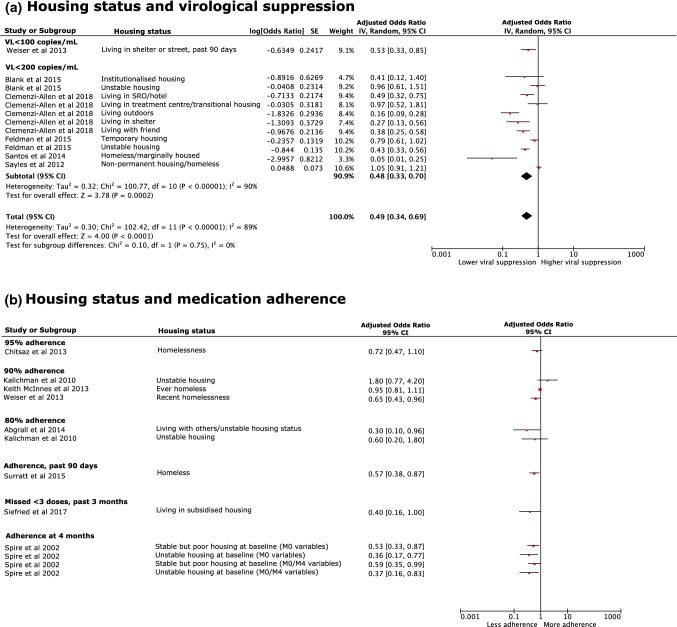
Forest plots of likelihood (aOR) of **a** virological suppression and **b** medication adherence among people living with HIV living in unstable, compared to stable, housing after adjusting for sociodemographic factors. A meta-analysis is presented for virological suppression. *M0* month 0, *M4* month 4, *SRO* single room occupancy, *VL* viral load

Of 83 studies, 39 looked at housing with eight studies reporting a significant adjusted association with virological suppression, all of which were a negative association, i.e., more unstable housing was associated with lower viral suppression [44, 48, 50, 53, 72, 85, 98, 103] (Fig. 4a; Additional file 6). As measurements and definitions of housing status and virological suppression were consistent, we conducted a random-effects meta-analysis of this determinant and outcome. Most included studies in the meta-analysis used a VL threshold < 200 copies/mL [46, 48, 53, 85, 86] however, one used < 100 copies/mL [98]. The pooled aOR of studies that used a VL threshold of < 200 copies/mL was 0.48 (95% CI 0.33, 0.70) with high heterogeneity between the studies (I^2^ = 90%). All but 5 reported subgroups found unstable housing to be significantly associated with lower viral suppression with the strongest relative effect reported by Santos et al. [85]. Santos et al*.* [85] found that homeless or marginally, compared to stably, housed transgender women living with HIV were 95% less likely (aOR 0.05; 95% CI 0.01, 0.25) to be virally suppressed after adjusting for social factors including age and race/ethnicity. Overall, the pooled aOR was 0.49 (95% CI 0.34, 0.69) with the random-effects model for meta-analysis displaying considerable heterogeneity across studies (I^2^ = 89%) [35].

Nine of 39 studies looking at housing found a significant adjusted association with medication adherence [45, 81, 94, 98, 102, 103, 113, 120, 122]; eight reported a negative association (unstable housing or homelessness) and one positive (long-term housing) (Fig. 4b; Additional file 6). Individuals living in unstable housing across a range of settings, including subsidised housing [122], living with others [102], being recently homeless [81, 94, 98, 113] or renting [103], were at greater risk of experiencing medication adherence failure, compared to those with more stable living situations. Spire et al. [120] looked at housing quality and found individuals living in stable, but poor housing or unstable housing at baseline have a greater likelihood of being non-adherent to HAART following 4-months of follow-up after adjusting for age, marital status and other intermediary determinants [120].

### Other Measures of Material Deprivation

**Fig. 5:**
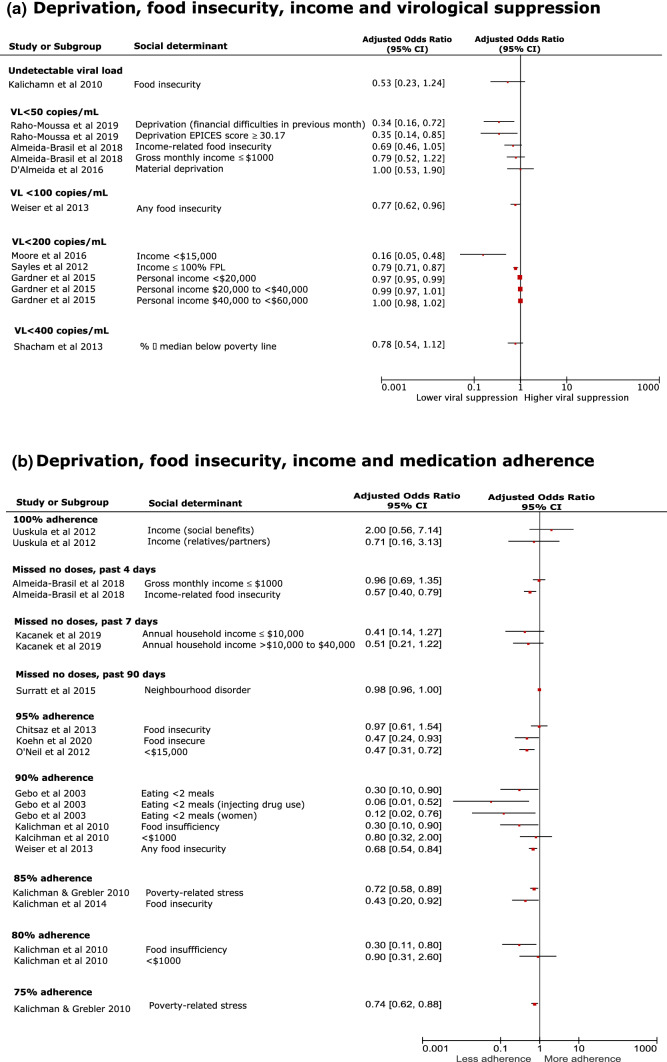
Forest plots of likelihood (aOR) of **a** virological suppression and **b** medication adherence among people living with HIV who were disadvantaged, compared to more advantaged after adjusting for sociodemographic factors. *EPICES* Evaluation of Deprivation and Inequalities in Health Examination Centres, *FPL* federal poverty level, *VL* viral load

Some studies grouped social determinants to report one overall deprivation measure; for instance, Kalichman and Grebler [63] grouped social determinants of housing, food and financial security as “poverty-related experiences” or “stressors.” We present comparable aORs exploring the association between measures of material deprivation, including food security, with virological suppression and medication adherence (Fig. 5; Additional file 6) and a narrative synthesis provided for each factor. Of 83 studies, 53 explored a measure of deprivation: specifically, food security (n = 7), poverty/deprivation explicitly (n = 9), income (n = 33) and socioeconomic status/position (n = 4).

#### Food Security

Food security is measured at the individual level using specific measurement tools such as the Household Food Security Survey Module (HFSSM). Food security was sometimes reported as a combined measure with income. One of seven food security studies reported a significant negative adjusted association with viral suppression [98]. Weiser et al.[98] found that individuals who reported any food insecurity were 29% more likely to be virologically non-suppressed (aOR 1.29; 95% CI 1.04, 1.61); however, the same study [98] found no difference in virological suppression among food insecure individuals after adjusting for adherence. Six studies investigating food security and adherence found a significant negative adjusted association [42, 56, 64, 65, 67, 98]. For instance, Almeida-Brasil et al.[42] examined a cohort of adults in Canada living with HIV and hepatitis C and established that there was no difference in virological suppression (VL > 50 copies/mL) among individuals reporting income-related food insecurity (aOR 1.44; 95% CI 0.95, 2.19) but were more likely to be non-adherent to medication (aOR 1.77; 95% CI 1.26, 2.48).

#### Poverty and Deprivation

Poverty and deprivation are reported as fundamentally neighbourhood or area level factors and based on context-specific measurement tools, including the Evaluation of Deprivation and Inequalities in Health Examination Centres (EPICES). Two of nine studies reported a significant adjusted negative association with virological suppression and deprivation [95, 115]. For instance, Raho-Moussa et al.[115] found that individuals who reported either individual determinants of deprivation (specifically financial difficulties in past month) or had an EPICES score indicating residence in a deprived state were 66% (aOR 0.34; 95% CI 0.16, 0.72) and 65% (aOR 0.35; 95% CI 0.14, 0.85) less likely, respectively, to achieve a VL < 50 copies/mL, compared to people living with HIV who did not meet these measures of deprivation, after adjusting for age and medication-, and clinically-related factors. Only one study reported a significant adjusted association with medication adherence which was in a negative direction [63]. Kalichman and Grebler [63] report that individuals reporting poverty-related stress are 28% (aOR 0.72; 95% CI 0.59, 0.89) and 26% (aOR 0.74; 95% CI 0.62, 0.88) less likely to report 85% and 75% ART adherence, respectively, after adjusting for social stressors, depression, internalised AIDS stigma and drug use.

#### Income

Income included components of temporality (e.g., annual), ecological level (e.g., household), sources (e.g., social benefits) and quantities (e.g., < $15,000). Four of 33 studies looking at income found a significant adjusted association with virological suppression [58, 77, 86, 101, 103]; three reported a negative association (lower income) and the other two a positive association (higher/mid-range income). For instance, the likelihood of virological non-suppression among Canadian men who have sex with men earning < $15,000 annually were 6-times greater (aOR 6.43; 95% CI 2.08, 19.89), than those earning more, after adjusting for age, ethnicity, sexual orientation, country of birth and other characteristics [77]. However, one study [58] found people living with HIV with an annual household income of < $10,000 were significantly more likely to report virological suppression, compared to those earning $10,000–$24,999 (aOR 0.21; 95% CI 0.06, 0.73) and $25,000-$49,999 (aOR 0.11; 95% CI 0.03, 0.52) [58]. The authors suggest that this may be due to those with lower annual household incomes being more likely to be receiving support linked to the Ryan White Program [58]. Sayles et al.[86] used federal poverty level (FPL) as a measure of income and found that uninsured people living with HIV in Los Angeles, who were receiving publicly funded healthcare through the Ryan White program, and had an income ≤ 100% FPL were 27% more likely (aOR 1.27; 95% CI 1.15, 1.41) to be virologically non-suppressed than those with an income greater than the FPL. Five studies reported a significant adjusted association between income and medication adherence [41, 57, 79, 96, 103]; negative associations were found among low income individuals [57, 79, 103] whilst, comparatively, mid-level/higher income were found to be positively associated [41, 96]. Vyas et al.[96] found individuals with a higher annual household income (≥ $10,000) were significantly more likely to be ≥ 90% ART adherent. The situation is less clear within the Veterans Aging Cohort Study whereby only individuals earning a mid-range annual household income ($25,000–$49,999) were significantly more likely to be adherent to medication, compared to those earning < $6000 [41]. A significant association was not found among the other 3 income categories [41]. Finally, Burch [103] reported whether individuals in the UK had financial stability through a proxy of having “enough money for basic needs”; they found that those who reported mostly, sometimes or not having enough were more likely to be non-adherent, compared to always having enough money.

#### Socioeconomic Status/Position (SES/SEP)

SES/SEP were reported across ecological levels. Two of four studies reported a significant positive adjusted association with higher/mid-range SES/SEP and viral suppression [87, 110]. Interestingly, a US study [87] found that individuals who had a “mid-range” SES which was defined by their payscale and whether they received support with healthcare costs (5–70% co-pay) were less likely (adjusted Relative Risk [aRR] 0.39; 95% CI 0.16, 0.94) to have a detectable HIV viral load, although this did not remain significant when considering CD4 counts < 200. Only one study looked at adjusted associations between SES and medication adherence; Parruti et al.[113] report no significant difference (OR 0.76; 95% CI 0.30, 2.00) in HAART adherence between people living with HIV in Italy with very low or low SES, compared to those with medium or high SES.

### Social Class

One study [114] explored the association between social class and treatment outcomes. Persson et al.[114] found no difference in the likelihood of having CD4 counts lower than the median value among skilled and unskilled workers (defined as social class III) compared to middle range civil servants (social class II) (OR 1.5; 95% CI 0.5, 4.9).

### Marginalised Sub-groups

Some studies followed specific subgroups of individuals who are often disproportionately affected by HIV; including, the homeless, people in prisons, people who use drugs and transgender women. Oftentimes, people will identify with multiple identities (intersectionality). For instance, Marshall et al. [72] reported that homeless people living with HIV who use drugs were almost half as likely (aPR 0.55; 95% CI 0.42, 0.71) to have an undetectable VL compared to those with housing, even after adjusting for sociodemographic factors and factors related to their substance use including addiction treatment. Berg et al. [45] found ART adherence rate was greater among individuals who were current or former opioid users who had lived in long-term housing even after adjusting for gender and intermediary determinants including alcohol and substance use. Finally, a small cross-section of transgender women living with HIV (n = 123) living in unstable housing had one of poorest virological outcomes, specifically their risk of having a detectable VL was over 7-times (aRR 7.37; 95% CI 1.07, 50.88) that of transgender women living in stable housing [44].

### Risk of Bias

Overall, 8 (9.6%) studies [51, 60, 69, 81, 102, 111, 117, 123] included in the review were scored ‘low quality’ or had a high risk of bias. Notably, several of the cross-sectional studies were possibly affected by selection bias; for instance, investigating a small sample size or not stating the frequency of non-respondents (Additional file 7). There was heterogeneous reporting within and between studies with no standardised approach for measuring and classifying social factors (exposures), outcomes or associations which limited possible study comparisons.

## Discussion

A small proportion (10–20%) of studies observed significant associations between material deprivation and poorer clinical outcomes. Overall, they suggest that people living with HIV who are the most materially deprived (housing, employment, deprivation, or income) display poorer viral suppression and medication adherence compared to those more advantaged. The strongest evidence is present for housing whereby 1 in 5 studies found unstable housing status was associated with poorer viral suppression; however, we found that 89% of the variability in the ORs could be explained by heterogeneity between the studies. An inconsistent association was observed for studies measuring education and adherence outcomes. A higher proportion of included studies observed significant, consistent associations between SES/SEP and virological suppression (50%) and food security and adherence (86%). We also found evidence that intersectionality worsens outcomes. The magnitude of associations were compounded within specific subgroups; for example, US transgender women who were homeless or marginally housed were 20-times more likely to be virologically non-suppressed than transgender women living in stable housing, after adjusting for age, race/ethnicity, history of injection drug use and health insurance status [85].

Our findings are in line with other studies including reviews which have found worsening outcomes associated with material deprivation, including unstable housing status, food insecurity and lower socioeconomic status [14, 124–126]. We find that these associations persist in high-income countries even as advanced generations of antiretrovirals become widely available, HIV prevention programmes continue to be scaled-up and HIV care evolves significantly through the digital age. For instance, in line with Krieger’s ecosocial theory [27, 28], issues of agency for HIV care exacerbate inequities among US women living with HIV [127]. This may be explained by the context-specific and interrelated nature of social determinants. For instance, school leavers ages vary across countries (from 15 to 18 years old) as does minimum wage, entitlements to welfare and state benefits and other support services available. In other words, social and cultural capital go beyond the measures of “relative deprivation” but can be more difficult to measure [128, 129]. Unlike Burch et al. [14], we did not explore health insurance as this is not a considerable barrier in countries with a universal healthcare system, such as the UK. Rather, factors such as social class drive health inequalities seen in the UK which were first described in the 1980 Black Report and later by Marmot’s Reports [3, 5, 130]. Associations between structural factors (HIV-related laws), interpersonal factors (perceived social capital) and individual outcomes (ART adherence) have also been identified across ecosocial context levels in North America which highlights the potential mechanisms of relationships between social and structural determinants [82]. Additionally, some determinants may be more directly associated with outcomes than others; for instance, some medication should be taken with food which may not be possible for somebody who is food insecure/insufficient, in turn influencing ART adherence [126].

The review had several strengths including a comprehensive search strategy and the inclusion of independent reviewers at screening, data extraction and quality assessment stages. We also grouped duplicate reports at the final stage of screening as one study and included the most recent report in the review to avoid possible publication bias. The majority of included studies were scored as low or uncertain risk of bias; however, the modified Newcastle–Ottawa scale used for cross-sectional studies has not been validated [37, 131]. Additionally, no grey literature was included, nor publications not published in English, which may have inadvertently excluded relevant studies, particularly from HIV community groups and charities. We only included observational studies, limiting the ability to make any causal inferences and potentially introducing social desirability bias of studies reliant on self-reported data only. Our review contributes to this field of knowledge but is unable to determine how determinants may or may not be causally linked but begins to suggest which determinants may interact with treatment outcomes of people living with HIV. We also recognise that social determinants are heavily interlinked, with collinearity between variables; however, we do not attempt to develop a causal pathway in this paper.

These findings re-emphasise the need for well-designed measures of social determinants in studies with evidence-based, context-specific definitions; for instance, higher-income countries (e.g., in Europe) use a ‘class structural’ approach to define occupation compared to low- and middle-income countries whereby occupation is highly dependent on working conditions (e.g., formal/informal sectors or environmental factors) [132]. As previously suggested by Krieger et al.[38] studies should be collecting socioeconomic data across all ecological levels and the lifecourse, treat income and poverty as dynamic, consider types of assets and wealth but also not conflate between SES and social class. We found that these recommendations were not met in most included studies and rather studies measured determinants at single timepoints or only at one ecological level. There is a clear gap for well-designed research looking at the impact of social class on treatment outcomes of people living with HIV. For instance, research into using subjective social class has found that individuals often use measures of socioeconomic position to assign status however, also take into account their current and future material and economic prospects which may be a more suitable, composite socioeconomic indicator [133].

Additionally, to develop a more conclusive understanding, a need remains to standardise thresholds of viral suppression across studies to allow for more detailed analyses of the direction of reported effect sizes. This would enable more robust random effects meta-analyses, and subsequent meta-synthesis, to be conducted despite issues relating to the generalisability of results and heterogeneous nature of studies. As a result, this would support the design of more appropriate, system-wide interventions and clinics to identify and support the most socioeconomically disadvantaged, and marginalised, people living with HIV. For instance, interventions focussing on the provision of stable and secure housing, rather than limiting this to adherence support for people continuing to live in precarious housing conditions which could have significant implications for HIV outcomes as well as wider mental health and wellbeing outcomes [134]. The Housing and Health Study for rental assistance found that HIV-related housing services were cost-effective as a HIV prevention intervention but also in relation to quality of life; a cost-per-QALY-saved estimated to be $62,493 for homeless and unstably housed people living with HIV across three US cities [135, 136]. At today’s exchange rate, this exceeds the UK National Institute for Health and Care Excellence (NICE) evidence-based guidelines for public health and social care services in for which as a cost-effectiveness threshold between £20,000-£30,000 per QALY gained [137].

Further research is required to determine to what extent person-centred and holistic HIV care improves the health and wellbeing outcomes among people living with HIV. For instance, the provision of social support services including housing, welfare and benefits advice, food and transport vouchers, alongside routine clinical monitoring to help address stigma, reduce health inequalities and ensure equitable access to HIV treatment and care.

## Conclusions

Our study has shown that the contribution of measures of material deprivation on HIV treatment outcomes remains complex. There is a need to propose a causal pathway of the relationship between these factors. An ecosocial analysis would help to establish the impact of social determinants between and across ecological levels (e.g., individual, household, neighbourhood) and how these are ‘embodied’ by individuals across the lifecourse. Further research might then be able to disentangle how social determinants are driven by inequalities in gender, sexual orientation, social class, and race/ethnicity allowing us to build a deeper understanding of how, when, and to what extent, these determinants interact with one another. Subsequently, this would ensure the support systems and services in places for the most marginalised groups living with HIV are acceptable and have the greatest impact on service users.

## Supplementary Information

Below is the link to the electronic supplementary material.

Supplementary file1 (PDF 70 kb)

Supplementary file2 (DOCX 36 kb)

Supplementary file3 (DOCX 19 kb)

Supplementary file4 (DOCX 21 kb)

Supplementary file5 (DOCX 35 kb)

Supplementary file6 (DOCX 221 kb)

Supplementary file7 (XLSX 19 kb)

## Data Availability

Supporting data are available from the corresponding author upon request.

## Abbreviations

aHR: Adjusted Hazard Ratio
aOR: Adjusted Odds Ratio
aPR: Adjusted Prevalence Ratio
aRR: Adjusted Relative Risk
ART: Antiretroviral therapy/treatment
cART: Combination antiretroviral therapy
CI: Confidence interval
CSDH: WHO Commission on Social Determinants of Health
EPICES: Evaluation of Deprivation and Inequalities in Health Examination Centres
FPL: Federal Poverty Level
HAART: Highly active antiretroviral therapy
HFSSM: Household Food Security Survey Module
MEMS: Medication Event Monitoring Systems
NICE: National Institute for Health and Care Excellence (UK)
OECD: Organisation for Economic Co-operation and Development
SEP/SES: Socioeconomic position/status
VL: Viral load
