# Supplementary material for: Influence of Material Deprivation on Clinical Outcomes Among People Living with HIV in High-Income Countries: A Systematic Review and Meta-analysis
Source: AIDS Behav. 2021 Dec 11;26(6):2026–54. doi: 10.1007/s10461-021-03551-y (PMC9046343; doi:10.1007/s10461-021-03551-y)
Supplement: Supplementary file 2 — Supplementary file2 (DOCX 36 kb) [file 10461_2021_3551_MOESM2_ESM.docx]

**Additional file 2: Search strategy**

Search strategy based on previous literature including previous PrEP/STI review [1], Cochrane review [2] and published systematic reviews focussing on HIV outcomes among people living with HIV (PLHIV) in high income countries (HICs) [3–5].

*Key concepts: HIV; social determinants/factors; HICs; and health/wellbeing outcomes*

**MEDLINE (Ovid)**

|  |  | Search terms (including MeSH) |
| --- | --- | --- |
| Population (person) | 1 | exp HIV infections/ |
|  | 2 | exp HIV/ |
|  | 3 | exp HIV Long-Term Survivors/ |
|  | 4 | (hiv OR hiv1 OR hiv2 OR human immun* deficiency virus* OR living with HIV).mp |
|  | 5 | 1 OR 2 OR 3 OR 4 |
| Exposure | 6 | exp Socioeconomic Factors/ |
|  | 7 | ((social OR socio?economic OR socio?demographic OR social economic) adj (determinant* OR factor* OR driver* OR status OR position* OR rank* OR class* OR capital OR condition* OR background* OR circumstance* OR depriv* OR disadvantage* OR inequal*)).mp. |
|  | 8 | 6 OR 7 |
| Population (setting) | 9 | exp Developed Countries/ |
|  | 10 | ((high?income OR developed) adj (countr* OR nation? OR population? OR world OR econom*)).mp |
|  | 11 | (Australia OR Austria OR Belgium OR Britain* OR Canada OR Chile OR Czech Republic OR Denmark OR England OR Estonia OR Finland OR France OR Germany OR Greece OR Hungary OR Iceland OR Ireland OR Irish Republic OR Israel OR Italy OR Japan OR Latvia OR Lithuania OR Luxembourg OR Netherlands OR New Zealand OR Northern Ireland OR Norway OR Poland OR Portugal OR Scotland OR Slovak Republic OR Slovakia OR Slovenia OR South Korea OR Korea Republic OR Spain OR Sweden OR Switzerland OR United Kingdom* OR United States OR Wales).mp. |
|  | 12 | 9 OR 10 OR 11 |
| Outcome | 13 | exp Antiretroviral Therapy, Highly Active/ |
|  | 14 | exp Health Status/ |
|  | 15 | exp Quality of Life/ |
|  | 16 | exp Patient Outcome Assessment/ |
|  | 17 | (anti?retroviral therap* OR anti?retroviral treatment* OR anti?retroviral drug* OR anti?retroviral regimen OR anti?retroviral adherence OR anti?retroviral compliance OR CD4* OR HIV vir* load* OR HIV vir* suppress* OR HIV* outcome* OR health* status OR health* outcome* OR health* inequal* OR quality of life* OR well?being OR patient* experience OR patient* outcome* OR HIV* care* OR HIV* treatment* OR HIV* service* OR HIV* pathway OR HIV* cascade).mp. |
|  | 18 | 13 OR 14 OR 15 OR 16 OR 17 |
|  | 19 | 5 AND 8 AND 12 and 18 |
| Search date: 13/01/2020  Results: 1074 | | |

**EMBASE (Ovid)**

|  | Search terms (including Emtree) |
| --- | --- |
| #1 | exp Human immunodeficiency virus/ |
| #2 | exp Human immunodeficiency virus infected patient/ |
| #3 | (hiv OR hiv1 OR hiv2 OR human immun* deficiency virus* OR living with HIV).mp |
| #4 | #1 OR #2 OR #3 |
| #5 | exp "social determinants of health"/ |
| #6 | exp socioeconomics/ |
| #7 | ((social OR socio?economic OR socio?demographic OR social economic) adj (determinant* OR factor* OR driver* OR status OR position* OR rank* OR class* OR capital OR condition* OR background* OR circumstance* OR depriv* OR disadvantage* OR inequal*)).mp. |
| #8 | #5 OR #6 OR #7 |
| #9 | exp high income country/ |
| #10 | exp developed country/ |
| #11 | ((high?income OR developed) adj (countr* OR nation? OR population? OR world OR econom*)).mp |
| #12 | (Australia OR Austria OR Belgium OR Britain* OR Canada OR Chile OR Czech Republic OR Denmark OR England OR Estonia OR Finland OR France OR Germany OR Greece OR Hungary OR Iceland OR Ireland OR Irish Republic OR Israel OR Italy OR Japan OR Latvia OR Lithuania OR Luxembourg OR Netherlands OR New Zealand OR Northern Ireland OR Norway OR Poland OR Portugal OR Scotland OR Slovak Republic OR Slovakia OR Slovenia OR South Korea OR Korea Republic OR Spain OR Sweden OR Switzerland OR United Kingdom* OR United States OR Wales).mp. |
| #13 | #9 OR #10 OR #11 OR #12 |
| #14 | exp antiretroviral therapy/ |
| #15 | exp health status/ |
| #16 | exp patient compliance/ |
| #17 | exp patient-reported outcome/ |
| #18 | exp "quality of life"/ |
| #19 | (anti?retroviral therap* OR anti?retroviral treatment* OR anti?retroviral drug* OR anti?retroviral regimen OR anti?retroviral adherence OR anti?retroviral compliance OR CD4* OR HIV vir* load* OR HIV vir* suppress* OR HIV* outcome* OR health* status OR health* outcome* OR health* inequal* OR quality of life* OR well?being OR patient* experience OR patient* outcome* OR HIV* care* OR HIV* treatment* OR HIV* service* OR HIV* pathway OR HIV* cascade).mp. |
| #20 | #14 OR #15 OR #16 OR #17 OR #18 OR #19 |
| #21 | #4 AND #8 AND #13 AND #20 |
| Search date: 13/01/2020  Results: 2079 | |

**Global Health (Ovid)**

|  | Search terms (including subject headings) |
| --- | --- |
| #1 | exp HIV infections/ |
| #2 | exp people living with HIV/ |
| #3 | (hiv OR hiv1 OR hiv2 OR human immun* deficiency virus* OR living with HIV).mp |
| #4 | #1 OR #2 OR #3 |
| #5 | exp socioeconomic status/ |
| #6 | exp socioeconomics/ |
| #7 | ((social OR socio?economic OR socio?demographic OR social economic) adj (determinant* OR factor* OR driver* OR status OR position* OR rank* OR class* OR capital OR condition* OR background* OR circumstance* OR depriv* OR disadvantage* OR inequal*)).mp. |
| #8 | #5 OR #6 OR #7 |
| #9 | exp Developed Countries/ |
| #10 | exp OECD Countries/ |
| #11 | ((high?income OR developed) adj (countr* OR nation? OR population? OR world OR econom*)).mp |
| #12 | (Australia OR Austria OR Belgium OR Britain* OR Canada OR Chile OR Czech Republic OR Denmark OR England OR Estonia OR Finland OR France OR Germany OR Greece OR Hungary OR Iceland OR Ireland OR Irish Republic OR Israel OR Italy OR Japan OR Latvia OR Lithuania OR Luxembourg OR Netherlands OR New Zealand OR Northern Ireland OR Norway OR Poland OR Portugal OR Scotland OR Slovak Republic OR Slovakia OR Slovenia OR South Korea OR Korea Republic OR Spain OR Sweden OR Switzerland OR United Kingdom* OR United States OR Wales).mp. |
| #13 | #9 OR #10 OR #11 OR #12 |
| #14 | exp highly active antiretroviral therapy/ |
| #15 | exp patient compliance/ |
| #16 | exp "quality of life"/ |
| #17 | (anti?retroviral therap* OR anti?retroviral treatment* OR anti?retroviral drug* OR anti?retroviral regimen OR anti?retroviral adherence OR anti?retroviral compliance OR CD4* OR HIV vir* load* OR HIV vir* suppress* OR HIV* outcome* OR health* status OR health* outcome* OR health* inequal* OR quality of life* OR well?being OR patient* experience OR patient* outcome* OR HIV* care* OR HIV* treatment* OR HIV* service* OR HIV* pathway OR HIV* cascade).mp. |
| #18 | #14 OR #15 OR #16 OR #17 |
| #19 | #4 AND #8 AND #13 AND #18 |
| Search date: 13/01/2020  Results: 682 | |

**HMIC Health Management Information Consortium (Ovid)**

|  | Search terms (including subject headings) |
| --- | --- |
| #1 | exp HIV/ |
| #2 | (hiv OR hiv1 OR hiv2 OR human immun* deficiency virus* OR living with HIV).mp |
| #3 | #1 OR #2 |
| #4 | exp socioeconomic factors/ |
| #5 | exp socioeconomic status/ |
| #6 | ((social OR socio?economic OR socio?demographic OR social economic) adj (determinant* OR factor* OR driver* OR status OR position* OR rank* OR class* OR capital OR condition* OR background* OR circumstance* OR depriv* OR disadvantage* OR inequal*)).mp. |
| #7 | #4 OR #5 OR #6 |
| #8 | exp Developed Countries/ |
| #9 | ((high?income OR developed) adj (countr* OR nation? OR population? OR world OR econom*)).mp |
| #10 | (Australia OR Austria OR Belgium OR Britain* OR Canada OR Chile OR Czech Republic OR Denmark OR England OR Estonia OR Finland OR France OR Germany OR Greece OR Hungary OR Iceland OR Ireland OR Irish Republic OR Israel OR Italy OR Japan OR Latvia OR Lithuania OR Luxembourg OR Netherlands OR New Zealand OR Northern Ireland OR Norway OR Poland OR Portugal OR Scotland OR Slovak Republic OR Slovakia OR Slovenia OR South Korea OR Korea Republic OR Spain OR Sweden OR Switzerland OR United Kingdom* OR United States OR Wales).mp. |
| #11 | #8 OR #9 OR #10 |
| #12 | exp health status/ |
| #13 | exp patient compliance/ |
| #14 | exp patient outcome/ |
| #15 | exp "quality of life"/ |
| #16 | (anti?retroviral therap* OR anti?retroviral treatment* OR anti?retroviral drug* OR anti?retroviral regimen OR anti?retroviral adherence OR anti?retroviral compliance OR CD4* OR HIV vir* load* OR HIV vir* suppress* OR HIV* outcome* OR health* status OR health* outcome* OR health* inequal* OR quality of life* OR well?being OR patient* experience OR patient* outcome* OR HIV* care* OR HIV* treatment* OR HIV* service* OR HIV* pathway OR HIV* cascade).mp. |
| #17 | #12 OR #13 OR #14 OR #15 OR #16 |
| #18 | #3 AND #7 AND #11 AND #17 |
| Search date: 13/01/2020  Results: 34 | |

**Cochrane Library**

|  | Search terms (including MeSH) |
| --- | --- |
| #1 | MeSH descriptor: [HIV Infections] explode all trees |
| #2 | MeSH descriptor: [HIV] explode all trees |
| #3 | MeSH descriptor: [HIV Long-Term Survivors] explode all trees |
| #4 | hiv OR hiv1 OR hiv2 OR human immun* deficiency virus* OR living with HIV:ab,ti,kw (Word variations have been searched) |
| #5 | #1 OR #2 OR #3 OR #4 |
| #6 | MeSH descriptor: [Socioeconomic Factors] explode all trees |
| #7 | social determinant* OR social factor* OR social driver* OR social status OR social position* OR social rank* OR socio?economic* OR social economic* OR socio?demographic* OR social class* OR social capital OR social condition* OR social background* OR social circumstance* OR social depriv* OR social disadvantage* OR social inequal*:ab,ti,kw (Word variations have been searched) |
| #8 | #6 OR #7 |
| #9 | MeSH descriptor: [Developed Countries] explode all trees |
| #10 | high?income countr* OR high?income nation? OR high?income population? OR developed nation? OR developed countr* OR developed world OR high?income group OR high?income econom* OR Australia OR Austria OR Belgium OR Britain* OR Canada OR Chile OR Czech Republic OR Denmark OR England OR Estonia OR Finland OR France OR Germany OR Greece OR Hungary OR Iceland OR Ireland OR Irish Republic OR Israel OR Italy OR Japan OR Latvia OR Lithuania OR Luxembourg OR Netherlands OR New Zealand OR Northern Ireland OR Norway OR Poland OR Portugal OR Scotland OR Slovak Republic OR Slovakia OR Slovenia OR South Korea OR Korea Republic OR Spain OR Sweden OR Switzerland OR United Kingdom OR United States* OR Wales:ab,ti,kw (Word variations have been searched) |
| #11 | #9 OR #10 |
| #12 | MeSH descriptor: [Antiretroviral Therapy, Highly Active] explode all trees |
| #13 | MeSH descriptor: [Health Status] explode all trees |
| #14 | MeSH descriptor: [Patient Outcome Assessment] explode all trees |
| #15 | MeSH descriptor: [Quality of Life] explode all trees |
| #16 | anti?retroviral therap* OR anti?retroviral treatment* OR anti?retroviral drug* OR anti?retroviral regimen OR anti?retroviral adherence OR anti?retroviral compliance OR CD4* OR HIV vir* load* OR HIV vir* suppress* OR HIV* outcome* OR health* status OR health* outcome* OR health* inequal* OR quality of life* OR well?being OR patient* experience OR patient* outcome* OR HIV* care* OR HIV* treatment* OR HIV* service* OR HIV* pathway OR HIV* cascade:ab,ti,kw (Word variations have been searched) |
| #17 | #12 OR #13 OR #14 OR #15 OR #16 |
| #18 | #5 AND #8 AND #11 AND #17 |
| Search date: 13/01/2020  Results: 62 | |

**CINAHL**

|  | Search terms (including subject headings) |
| --- | --- |
| S1 | (MH “Human Immunodeficiency Virus+”) |
| S2 | (MH “HIV-Infected Patients+”) |
| S3 | (hiv OR hiv1 OR hiv2 OR “human immun* deficiency virus*” OR “living with HIV”) |
| S4 | S1 OR S2 OR S3 |
| S5 | (MM “Social Determinants of Health”) |
| S6 | (MH “Socioeconomic Factors+” |
| S7 | (“social determinant*” OR “social factor?” OR “social driver?” OR “social status” OR “social position*” OR “social rank*” OR “socio#economic*” OR “social economic*” OR socio#demographic* OR “social class*” OR “social capital” OR “social condition?” OR “social background?” OR “social circumstance?” OR “social depriv*” OR “social disadvantage*” OR “social inequal*”) |
| S8 | S5 OR S6 OR S7 |
| S9 | (MM “Developed Countries”) |
| S10 | (“high?income countr*” OR “high?income nation?” OR “developed nation?” OR “developed countr*” OR “developed world” OR “high?income group?” OR “high?income population?” OR “high?income econom*” OR Australia OR Austria OR Britain* OR Belgium OR Canada OR Chile OR “Czech Republic” OR Denmark OR England OR Estonia OR Finland OR France OR Germany OR Greece OR Hungary OR Iceland OR Ireland OR “Irish Republic” OR Israel OR Italy OR Japan OR Latvia OR Lithuania OR Luxembourg OR Netherlands OR “New Zealand” OR “Northern Ireland” OR Norway OR Poland OR Portugal OR Scotland OR “Slovak Republic” OR Slovakia OR Slovenia OR “South Korea” OR “Korea Republic” OR Spain OR Sweden OR Switzerland OR “United Kingdom” OR “United States*” OR Wales) |
| S11 | S9 OR S10 |
| S12 | (MM "Antiretroviral Therapy, Highly Active") |
| S13 | (MH “Health Status+”) |
| S14 | (MH “Quality of Life+”) |
| S15 | (MM "Outcome Assessment") |
| S16 | (“anti?retroviral therap*” OR “anti?retroviral treatment*” OR “anti?retroviral* drug*” OR “anti?retroviral regimen” OR “anti?retroviral adherence” OR “anti?retroviral compliance” OR CD4* OR “HIV vir* load*” OR “HIV vir* suppress*” OR “HIV* outcome*” OR “health status” OR “health* outcome*” OR “health inequal*” OR “quality of life*” OR well#being OR “patient* experience” OR “patient* outcome*” OR “HIV* care*” OR “HIV* treatment*” OR “HIV* service*” OR “HIV* pathway” OR “HIV* cascade") |
| S17 | S12 OR S12 OR S13 OR S14 OR S15 OR S16 |
| S18 | S4 AND S8 AND S11 AND S17 |
| Search date: 13/01/2020  Results: 708 | |

**Scopus**

| Search terms |
| --- |
| ( ( TITLE-ABS-KEY ( hiv OR hiv1 OR hiv2 OR "human immunodeficiency virus" OR "living with HIV" ) ) AND ( ( TITLE-ABS-KEY ( "social determinant*" OR "social factor*" OR "social driver*" OR "social status" OR "social position*" OR "social rank*" OR socio?economic* OR "social economic*" OR socio?demographic* ) ) OR ( TITLE-ABS-KEY ( "social class*" OR "social capital" OR "social condition*" OR "social background*" OR "social circumstance*" OR "social depriv*" OR "social disadvantage*" OR “social inequal*” ) ) ) AND ( ( TITLE-ABS-KEY ( "high?income countr*" OR "high?income nation" OR "developed nation?" OR "developed countr*" OR “developed world” OR "high?income group" OR “high?income population*” OR "high?income econom*" ) ) OR ( TITLE-ABS-KEY (australia OR austria OR belgium OR britain* OR canada OR chile OR "czech republic" OR denmark OR england OR estonia OR france OR finland OR germany OR greece OR hungary OR iceland OR ireland OR "irish republic" OR israel OR italy OR japan OR latvia OR lithuania OR luxembourg OR netherlands) ) OR ( TITLE-ABS-KEY ( "New Zealand" OR norway OR “Northern Ireland” OR poland OR portugal OR scotland OR slovakia OR “slovak republic” OR slovenia OR "south korea" OR "korea republic" OR spain OR sweden OR switzerland OR "united kingdom" OR "united states*" OR wales ) ) ) AND ( ( TITLE-ABS-KEY ( "anti?retroviral therap*" OR “anti?retroviral treatment*” OR "anti?retroviral drug*" OR "anti?retroviral regimen" OR "anti?retroviral adherence" OR "anti?retroviral compliance" OR "highly active anti?retroviral*" OR cd4* OR "HIV vir* load" OR "HIV vir* suppress*" OR "HIV* outcome*" ) ) OR ( TITLE-ABS-KEY ( "health status" OR “health* outcome*” OR “health inequal*” OR "quality of life*" OR well?being OR “patient* experience” OR "patient* outcome*" ) ) OR ( TITLE-ABS-KEY ( "HIV* care*" OR "HIV* treatment*" OR "HIV* service*” OR “HIV* pathway” OR “HIV* cascade" ) ) ) ) |
| Search date: 13/01/2020  Results: 475 |

**Web of Science**

| Search terms |
| --- |
| \| TS=(((hiv OR hiv1 OR hiv2 OR human immun* deficiency virus* OR “living with HIV”))) AND TS=(((“social determinant*” OR “social factor$” OR “social driver$” OR “social status” OR “social position*” OR “social rank*” OR socio?economic* OR “social economic*” OR socio?demographic* OR “social class*” OR “social capital” OR “social condition*” OR “social background$” OR “social circumstance$” OR “social depriv*” OR “social disadvantage$” OR “social inequal*”))) AND TS=(((“high?income countr*” OR “high?income nation$” OR “developed nation$” OR “developed countr*” OR “developed world” OR “high?income group$” OR “high?income population$” OR “high?income econom*” OR Australia OR Austria OR Belgium OR Britain* OR Canada OR Chile OR “Czech Republic” OR Denmark OR England OR Estonia OR Finland OR France OR Germany OR Greece OR Hungary OR Iceland OR Ireland OR “Irish Republic” OR Israel OR Italy OR Japan OR Latvia OR Lithuania OR Luxembourg OR Netherlands OR “New Zealand” OR “Northern Ireland” OR Norway OR Poland OR Portugal OR “Slovak Republic” OR Slovakia OR Slovenia OR “South Korea” OR “Korea Republic” OR Scotland OR Spain OR Sweden OR Switzerland OR “United Kingdom” OR “United States*” OR Wales))) AND TS=(((“anti?retroviral therap*” OR “anti?retroviral treatment*” OR “anti?retroviral drug*” OR “anti?retroviral regimen” OR “anti?retroviral adherence” OR “anti?retroviral compliance” OR CD4* OR “HIV vir* load*” OR “HIV vir* suppress*” OR “HIV* outcome$” OR “health status” OR “health* outcome” OR “health inequal*” OR “quality of life*” OR well?being OR “patient* experience” OR “patient* outcome$” OR “HIV* care*” OR “HIV* treatment*” OR “HIV* service*” OR “HIV* pathway” OR “HIV* cascade" )))  Indexes=SCI-EXPANDED, SSCI, A&HCI, CPCI-S, CPCI-SSH, ESCI Timespan=All years \|  \| \| --- \| --- \| |
| Search date: 13/01/2020  Results: 130 |

**ProQuest**

| Search terms (including index terms) |
| --- |
| (diskw(HIV) OR diskw("People living with HIV") OR noft(hiv OR hiv1 OR hiv2 OR "human immun* deficiency virus*" OR "living with HIV")) AND (diskw("Social determinants of health") OR diskw("Socioeconomic status") OR noft(("social determinants") OR ("social factor" OR "social factors") OR "social driver" OR "social status" OR ("social position" OR "social positioning" OR "social positions") OR ("social rank" OR "social ranking" OR "social ranks") OR socio#economic* OR social economic* OR socio#demographic OR ("social class" OR "social classes" OR "social classification" OR "social classifications") OR "social capital" OR ("social condition" OR "social conditioning" OR "social conditions") OR "social background" OR ("social circumstance" OR "social circumstances") OR ("social deprivation") OR ("social disadvantage" OR "social disadvantages") OR ("social inequalities" OR "social inequality"))) AND noft("high?income countr*" OR "high?income nation?" OR "developed nation?" OR ("developed countries" OR "developed country") OR "high?income group" OR "high?income econom*" OR Australia OR Austria OR Belgium OR Britain OR Canada OR Chile OR "Czech Republic" OR Denmark OR England OR Estonia OR Finland OR France OR Germany OR Greece OR Hungary OR Iceland OR Ireland OR "Irish Republic" OR Israel OR Italy OR Japan OR Latvia OR Lithuania OR Luxembourg OR Netherlands OR "New Zealand" OR "Northern Ireland" OR Norway OR Poland OR Portugal OR Scotland OR "Slovak Republic" OR Slovakia OR Slovenia OR "South Korea" OR "Korea Republic" OR Spain OR Sweden OR Switzerland OR "United Kingdom" OR ("united states" OR "united statesa") OR Wales) AND (diskw("Antiretroviral therapy") OR diskw("Health outcomes") OR diskw("Health status") OR diskw("Quality of life") OR noft((("anti?retroviral therap*" OR "anti?retroviral treatment*" OR "anti?retroviral* drug*" OR "anti?retroviral regimen" OR "anti?retrovial adherence" OR "anti?retroviral compliance" OR CD4* OR "HIV vir* load*" OR "HIV vir* suppress*" OR "HIV* outcome*" OR "health status" OR "health* outcome*" OR "health inequal*" OR "quality of life*" OR well#being OR "patient* experience" OR "patient* outcome*" OR "HIV* care*" OR "HIV* treatment*" OR "HIV* service*" OR "HIV* pathway" OR "HIV* cascade")))) |
| Search date: 13/01/2020  Results: 326 |

**Hand searches**

| **Source** | **Search date** | **Studies added to screening** |
| --- | --- | --- |
| Snowballing from 8 reviews and 9 editorials |  | 45 |
| Articles identified previously by me during reading | October 2019 to March 2020 | 9 |
| CROI conference database   - *Search terms: social determinant, social factor* - *Conference dates: 2014 to 2019* | 9/03/2020 | 2 |
| BHIVA conference database   - *Search terms: social determinant, social factor* - *Conference dates: 2009 to 2019* | 9/03/2020 | 0 |
| AIDS Impact conference database   - *Search terms: social determinant, social factor* - *Conference dates: 2007 to 2019* | 9/03/2020 | 2 |
| Abstract Archive IAS conference database   - *Search terms: social determinant* | 9/03/2020 | 3 |

Studies found were searched against those already picked up in database searches. Any duplicates were removed before then being added to screening.

**References**

1. Papageorgiou V, Crittendon E, Davies B, Ward H. Impact of pre-exposure prophylaxis (PrEP) on the risk of bacterial sexually transmitted infections (STIs) among cisgender women: a systematic review [Internet]. PROSPERO. 2019 [cited 2019 Dec 11]. Available from: https://www.crd.york.ac.uk/PROSPERO/display_record.php?RecordID=130438

2. Li L, Smith H, Atun R, Tudor Car L. Search strategies to identify observational studies in MEDLINE and Embase. Cochrane Database of Systematic Reviews [Internet]. 2019;(3). Available from: https://doi.org//10.1002/14651858.MR000041.pub2

3. McAllister A, Fritzell S, Almroth M, Harber-Aschan L, Larsson S, Burström B. How do macro-level structural determinants affect inequalities in mental health? – a systematic review of the literature. International Journal for Equity in Health. 2018 Dec 6;17(1):180.

4. Alvarez-del Arco D, Monge S, Azcoaga A, Rio I, Hernando V, Gonzalez C, et al. HIV testing and counselling for migrant populations living in high-income countries: a systematic review. European Journal of Public Health. 2012 Sep 23;23(6):1039–45.

5. Burch LS, Smith CJ, Phillips AN, Johnson MA, Lampe FC. Socioeconomic status and response to antiretroviral therapy in high-income countries: a literature review. Aids. 2016;30(8):1147–62.
