# Supplementary material for: Influence of Material Deprivation on Clinical Outcomes Among People Living with HIV in High-Income Countries: A Systematic Review and Meta-analysis
Source: AIDS Behav. 2021 Dec 11;26(6):2026–54. doi: 10.1007/s10461-021-03551-y (PMC9046343; doi:10.1007/s10461-021-03551-y)
Supplement: Supplementary file 3 — Supplementary file3 (DOCX 19 kb) [file 10461_2021_3551_MOESM3_ESM.docx]

**Additional file 3: Eligibility criteria for study inclusion**

| **Inclusion criteria** | **Exclusion criteria** |
| --- | --- |
| - **Study design:** Quantitative studies observable in real-world setting (observational studies; e.g. cross-sectional, cohort, longitudinal). - **Publication:** Full-text, peer-reviewed journal articles, conference abstracts or grey literature published in English. - **Geographic location of studies:** high-income countries. - **Publication date:** not limited (but note whether before or after introduction of ART in country). - **Publication status:** published and in progress. | - **Study design:** interventional studies (e.g. RCTs) and qualitative studies (e.g. interviews, focus groups) and case studies. - **Geographic location of studies:** low and middle-income countries - **Exclude** studies where social factors are presented as outcomes of HIV rather than determinants. - **Exclude** studies focussing on HIV testing, transmission (i.e. risk of infection), disclosure of HIV status and/or prevention rather than the experiences/health/wellbeing of people living with HIV who are aware of their status etc. - **Exclude** studies that do not include primary outcome(s) of ART adherence and/or measures of viral suppression. - **Exclude** studies not meeting PECO criteria (e.g. focussing on outcomes such as mortality, HIV-related symptoms, co-morbidities, hospitalisations etc.) |
