# Supplementary material for: Influence of Material Deprivation on Clinical Outcomes Among People Living with HIV in High-Income Countries: A Systematic Review and Meta-analysis
Source: AIDS Behav. 2021 Dec 11;26(6):2026–54. doi: 10.1007/s10461-021-03551-y (PMC9046343; doi:10.1007/s10461-021-03551-y)
Supplement: Supplementary file 4 — Supplementary file4 (DOCX 21 kb) [file 10461_2021_3551_MOESM4_ESM.docx]

**Additional file 4: Data extraction table**

| Study | - Author - Year - Title - Publication type - Funding source(s) - Potential conflicts of interest |
| --- | --- |
| Study characteristics | - Aim/objective(s) - Study design - Population - Recruitment period/date of analysis - Setting - Eligibility criteria - Time - Unit of analysis and statistical methods used |
| Exposure (social determinant) | - Variable name - Measurement instrument and definition - Method of aggregation (e.g. mean, SD, %) - Timing of measurement |
| HIV outcome | - Variable name - Measurement instrument and definition - Method of aggregation (e.g. mean, SD, %) - Timing of measurement |
| Results | - Key finding: association between exposure(s) and outcome(s), e.g. RR, OR, PRR (adjusted and unadjusted) |
| Miscellaneous | - Impressions - Context (e.g. correspondence to author) |

OR: odds ratio; PRR: prevalence rate ratio; RR: relative risk; SD: standard deviation

Table adapted from Chapter 5.3.1a and 5.3.5, Cochrane Handbook [1]

**References**

1. Li T, Higgins J, Deeks J. Chapter 5: Collecting data. In: Higgins J, Thomas J, Chandler J, Cumpston M, Li T, Page M, et al., editors. Cochrane Handbook for Systematic Reviews of Interventions version 60 (updated July 2019) [Internet]. Cochrane; 2019 [cited 2020 Mar 24]. Available from: www.training.cochrane.org/handbook
