# Supplementary material for: Influence of Material Deprivation on Clinical Outcomes Among People Living with HIV in High-Income Countries: A Systematic Review and Meta-analysis
Source: AIDS Behav. 2021 Dec 11;26(6):2026–54. doi: 10.1007/s10461-021-03551-y (PMC9046343; doi:10.1007/s10461-021-03551-y)
Supplement: Supplementary file 5 — Supplementary file5 (DOCX 35 kb) [file 10461_2021_3551_MOESM5_ESM.docx]

**Additional file 5: Newcastle-Ottawa Scale – data abstraction forms**

Tables adapted from original Newcastle-Ottawa Scale [1] and Anglin *et al* [2].

| **NEWCASTLE - OTTAWA QUALITY ASSESSMENT SCALE: COHORT / LONGITUDINAL STUDIES (out of 9)** | | | | |
| --- | --- | --- | --- | --- |
| **Bias** | **Cohort** | **High Quality** |  |  |
| **SELECTION**  **(max ⋆⋆⋆⋆)** | Representativeness of the exposed cohort  **(adults 18+ living with HIV and aware of their status)** | Truly representative of average in target population **⋆**  Somewhat representative of average in target population **⋆** | Selected group of users *(e.g. particular occupation)* | No description of derivation of cohort |
|  | Selection of the non-exposed cohort | Drawn from the same community as the exposed cohort **⋆** | Drawn from a different source | No description of the derivation of the non-exposed cohort |
|  | Ascertainment of exposure | Secure record (e.g. surgical, laboratory or medical records) **⋆**  Structured interview **⋆** | Self-report | No description |
|  | Demonstration that outcome of interest was not present at start of study | Yes **⋆**  *Note: statement of no history of disease or incident earns a star.* | No |  |
| **COMPARABILITY (max. ⋆⋆)** | Comparability of cohorts on the basis of the design or analysis  *Note: may be multiple ratings for different categories of exposure (e.g. ever vs never)* | Study controls for **at least gender, sexual identity or age** (or analysis separated by factors) **⋆**  Study controls for **race/ethnicity** and/or **social class ⋆** | Fails to control for an important factor  Does not control for any factors | *Statements of no differences between groups/not statistically significant not sufficient for establishing comparability.* |
| **OUTCOME**  **(max. ⋆⋆⋆)** | Assessment of outcome | Independent or blind assessment **⋆** *(i.e. stated in paper or confirmation of outcome by reference to secure records e.g. medical records, electronic monitoring)*  Record linkage **⋆** *(e.g. ICD codes on database record or electronic medical record)* | Self-report (*i.e. no reference to original medical records to confirm outcome)* | No description |
|  | Was follow-up long enough for outcomes to occur | Yes **[viral suppression/viral load/CD4 count (at least 6 months)[**3]**; ART adherence (minimum of 30 days)**[4]] **⋆** | No |  |
|  | Adequacy of follow up of cohorts | Complete follow up - all subjects accounted for **⋆**  Subjects lost to follow up unlikely to introduce bias: small number lost (**<20%)** to follow up, or description provided of those lost)**⋆** [5,6] | Follow up rate <**80%** and no description of those lost | No statement |

Loss to follow-up was calculated using the “standard percentage method” despite this not accounting for individual person-time contributed [7].

| **NEWCASTLE - OTTAWA QUALITY ASSESSMENT SCALE (adapted for cross-sectional studies) (out of 8)** | | | | |
| --- | --- | --- | --- | --- |
| **Bias** | **Cross-sectional study** | **High Quality / Low risk of bias** |  |  |
| **SELECTION**  **(max. ⋆⋆⋆⋆)** | Representativeness of the sample | Truly representative of the average in the target population. **⋆** (all subjects or random sampling)  Somewhat representative of the average in the target population. **⋆** (non-random sampling) | Selected group of users *(e.g. particular occupation)* | No description of the sampling strategy |
|  | Sample size | Justified and satisfactory **⋆** | Not justified |  |
|  | Non-respondents | Comparability between respondents and non-respondents’ characteristics is established, and the response rate is satisfactory (>60%). **⋆** | The response rate is unsatisfactory, or the comparability between respondents and non-respondents is unsatisfactory. | No description of the response rate or the characteristics of the responders and the non-responders. |
|  | Ascertainment of exposure (risk factor) | Validated measurement tool / secure record **⋆** | Non-validated measurement tool, but the tool is available or described. **⋆** | No description of the measurement tool. |
| **COMPARABILITY (max. ⋆⋆)** | Comparability of subjects in different outcome groups on the basis of the design or analysis. Confounding factors are controlled. | study controls for **at least gender, sexual identity or age** (or analysis separated by factors) **⋆**  study controls for **race/ethnicity** and/or **social class ⋆** | Fails to control for an important factor  Does not control for any factors |  |
| **OUTCOME**  **(max. ⋆⋆)** | Assessment of outcome | Independent or blind assessment **⋆**  *[Note: stated in paper, or confirmation of the outcome by reference to secure records (e.g. medical records or electronic monitoring)]*  Record linkage **⋆**  *(e.g. ICD codes on database record or electronic medical record)* | Self-report *i.e. no reference to original medical records to confirm outcome* | No description |
|  | Statistical test | The statistical test used to analyse the data is clearly described and appropriate, and the measurement of the association is presented, including confidence intervals and the probability level (p value). **⋆** | The statistical test is not appropriate or incomplete. | The statistical test is not described. |

Newcastle-Ottawa Scale (cross-sectional) adapted from Modesti *et al* [8] – similarly, we did not select a single variable to measure for comparability as variables differed across studies. However, we did not assign any stars to self-reported measures, nor gave 2 stars for studies that use independent observers / record linkage to assess outcomes. If the survey response rate not provided, this was calculated using the American Association for Public Opinion Research response rate 1 (AAPOR RR1 formula) [9].

**References**

1. Wells GA, Shea B, O’connell D, Peterson J, Welch V, Losos M, et al. The Newcastle-Ottawa Scale (NOS) for assessing the quality of nonrandomised studies in meta-analyses. 2013. 2016; Available from: http://www.ohri.ca/programs/clinical_epidemiology/oxford.asp

2. Anglin RES, Samaan Z, Walter SD, McDonald SD. Vitamin D deficiency and depression in adults: systematic review and meta-analysis. British Journal of Psychiatry. 2013;202(2):100–7.

3. World Health Organization. Consolidated guidelines on the use of antiretroviral drugs for treating and preventing HIV infection: recommendations for a public health approach. World Health Organization; 2016.

4. World Health Organization. Patient monitoring guidelines for HIV care and antiretroviral therapy (ART). 2006;

5. Hartling L, Hamm M, Milne A, Vandermeer B, Santaguida PL, Ansari M, et al. Appendix E, Decision Rules for Application of the Newcastle-Ottawa Scale. In: Validity and inter-rater reliability testing of quality assessment instruments [Internet]. Agency for Healthcare Research and Quality (US); 2012 [cited 2020 Jul 3]. Available from: https://www.ncbi.nlm.nih.gov/books/NBK92291/

6. Kristman V, Manno M, Côté P. Loss to Follow-Up in Cohort Studies: How Much is Too Much? European Journal of Epidemiology. 2004 Aug 1;19(8):751–60.

7. Xue X, Agalliu I, Kim MY, Wang T, Lin J, Ghavamian R, et al. New methods for estimating follow-up rates in cohort studies. BMC Medical Research Methodology. 2017 Dec 1;17(1):155.

8. Modesti PA, Reboldi G, Cappuccio FP, Agyemang C, Remuzzi G, Rapi S, et al. Panethnic differences in blood pressure in Europe: a systematic review and meta-analysis. PloS one. 2016;11(1):e0147601.

9. American Association for Public Opinion Research. Response Rates - An Overview [Internet]. [cited 2020 Jul 12]. Available from: https://www.aapor.org/Education-Resources/For-Researchers/Poll-Survey-FAQ/Response-Rates-An-Overview.aspx
