# Supplementary material for: Influence of Material Deprivation on Clinical Outcomes Among People Living with HIV in High-Income Countries: A Systematic Review and Meta-analysis
Source: AIDS Behav. 2021 Dec 11;26(6):2026–54. doi: 10.1007/s10461-021-03551-y (PMC9046343; doi:10.1007/s10461-021-03551-y)
Supplement: Supplementary file 6 — Supplementary file6 (DOCX 221 kb) [file 10461_2021_3551_MOESM6_ESM.docx]

**Outcome 1: Virological (non-) suppression**

| **Author** | **Population (country)** | **Measure of social determinant** | **Main unadjusted results estimate (95% CI)*** | **Main adjusted results estimate (95% CI)****^[[1]](#endnote-1)^** |
| --- | --- | --- | --- | --- |
| Almeida-Brasil et al 2018 [1] | 566 adults living with HIV and hepatitis C receiving cART treatment (Canada) | **Educational** level (ref: ≤high school education)  **Employment** status (ref: not working)  Income-related **food insecurity** (defined as Household Food Security Survey Module, HFSSM, score ≥2)  Gross monthly **income** $1000CAD or less | VL >50 copies/mL  >High school education (i.e. College or University): OR 1.51 (0.90, 2.53)  Working: OR 1.10 (0.65, 1.86) | *Adjusted for age, sex, housing status, employment status, income etc*  VL >50 copies/mL  Food insecurity: aOR 1.44 (0.95, 2.19)  Income: aOR 1.26 (0.82, 1.93) |
| Anderson et al 2018 [2] | 239 adult women living with HIV at an urban HIV specialty clinic (USA) | **Educational** level (ref: <high school graduation)  **Employment** status (ref: unemployed) | VL>20 copies/mL  ≥High school graduation: **OR 0.375 (0.204, 0.690); p=0.002**  Employed: OR 0.414 (0.137, 1.247); p=0.117  CD4 count <200 cell/mm^3^  ≥High school graduation: OR 1.265 (0.528, 3.029); p=0.598  Employed: OR 1.458 (0.459, 4.63); p=0.522 | *Adjusted for age, educational level, employment status, alcohol/drug use, mental health outcomes and past year intimate partner violence (and viral suppression)*  VL>20 copies/mL  ≥High school graduation: **aOR 0.367 (0.185, 0.728); p=0.004**  Employed: aOR 0.435 (0.121, 1.567); p=0.203  CD4 count <200 cell/mm^3^  ≥High school graduation: aOR 2.415 (0.793, 7.355); p=0.121  Employed: aOR 3.174 (0.69, 14.599); p=0.138 |
| Baguso et al 2019 [3] | 123 self-identified transwomen (USA) | **Educational** level (ref: ≤high school)  Unstable **housing** (yes, ref: no) | Unknown viral load  High school diploma: RR 0.91 (0.23, 3.53); p=0.891  >High school: RR 1.52 (0.38, 6.02); p=0.548  Unstable housing: RR 1.20 (0.38, 3,76); p=0.757.  Detectable viral load  High school diploma: RR 0.93 (0.15, 5.80); p=0.937  >High school: RR 0.95 (0.17, 5.27); p=0.951  Unstable housing: **RR 6.42 (1.27, 32.48)** | *Adjusted for age, gender identity, race/ethnicity, education, born in US and intermediary factors (e.g. discrimination)*  Unknown viral load  Unstable housing: ARR 0.71 (0.20, 2.50); p=0.595  Detectable viral load  Unstable housing: **ARR 7.37 (1.07, 50.88); p=0.043** |
| Blank et al 2015 [4] | 944 women of colour living with HIV (USA) | **Education** (ref: <high school)  **Employment** status (ref: working part-/full-time)  **Housing status** (ref: stable) |  | *Adjusted for age, race/ethnicity, education, migrant status, health status, substance use, sexual behaviour, intimate partner violence*  VL <200 copies/mL  ≥High school graduate or higher: aOR 1.29 (0.67, 2.49)  Not working/other employment status: aOR 0.836 (0.41, 1.69)  Disabled employment status: aOR 0.695 (0.33, 1.45)  Institutionalised housing: aOR 0.409 (0.12, 1.38)  Unstably housed: aOR 0.964 (0.61, 1.52) |
| Burch 2018 [5] | 2704 individuals living with HIV recruited from eight HIV outpatient clinics (UK) | University **education**  **Employment**  **Housing** status (ref: homeowner)  Enough **money** for basic needs? (financial hardship) (ref: always) | VL >50 copies/mL  No university education: **PR 1.63 (1.23, 2.16); p=0.0004**  Unemployed: **PR 1.85 (1.42, 2.41); p<0.0001**  Renting housing status: **PR 2.39 (1.69, 3.39)**  Unstable housing status: **PR 3.70 (2.42, 5.67)**  Enough money for basic needs? Mostly: **PR 1.63 (1.15, 2.3)**  Enough money for basic needs? Sometimes: **PR 2.06 (1.44, 2.95)**  Enough money for basic needs? No: **PR 2.68 (1.87, 3.86)**  VL >200 copies/mL  No university education: **HR 1.52 [rate 4.24] (1.07, 2.17); p=0.021**  Unemployed: **HR 2.56 [rate 5.78] (1.81, 3.62); p<0.0001**  Renting housing status: **HR 2.80 [rate 4.76] (1.81, 4.32); p<0.0001**  Unstable housing status: **HR 4.11 [rate 6.95] (2.27, 7.42); p<0.0001**  Enough money for basic needs? Mostly: HR 1.47 (0.95, 2.27)  Enough money for basic needs? Sometimes: **HR 2.25 (1.43, 3.55)**  Enough money for basic needs? No: **HR 2.78 (1.71, 4.53)** | *Adjusted for gender/sexual orientation and age.*  VL >50 copies/mL  No university education: **aPR 1.62 (1.22, 2.14); p=0.0005**  Unemployed: **aPR 1.98 (1.51, 2.61); p<0.0001**  Renting housing status: **aPR 2.12 (1.49, 3.02); p<0.0001**  Unstable housing status: **aPR 3.04 (1.97, 4.68); p<0.0001**  Enough money for basic needs? Mostly: **aPR 1.57 (1.11, 2.22)**  Enough money for basic needs? Sometimes: **aPR 1.87 (1.29, 2.72)**  Enough money for basic needs? No: **aPR 2.42 (1.67, 3.51)**  VL >200 copies/mL  No university education: **aHR 1.50 (1.05, 2.15); p=0.025**  Unemployed: **aHR 2.87 (2.00, 4.10); p<0.0001**  Renting housing status: **aHR 2.33 (1.49. 3.65); p<0.0001**  Unstable housing status: **aHR 3.18 (1.73, 5.85); p<0.0001**  Enough money for basic needs? Mostly: aHR 1.34 (0.87, 2.09)  Enough money for basic needs? Sometimes: **aHR 1.83 (1.14, 2.95)**  Enough money for basic needs? No: **aHR 2.30 (1.39, 3.81)** |
| Clemenzi-Allen et al 2018 [6] | 1222 people living with HIV at HIV clinic (USA) | **Housing status** (ref: rent/own) | VL <200 copies/mL  Living in treatment centre/transitional housing: OR 0.74 (0.40, 1.36); p=0.33  Living in Single Room Occupancy (SRO)/hotel: **OR 0.40 (0.26, 0.60); p<0.001**  Living with friend: **OR 0.30 (0.20, 0.45); p<0.001**  Living in shelter: **OR 0.25 (0.12, 0.53); p<0.001**  Living outdoors: **OR 0.12 (0.07, 0.23); p<0.001** | *Adjusted for age, race/ethnicity, gender*  VL <200 copies/mL  Treatment centre/transitional housing: aOR 0.97 (0.52, 1.81) p=0.76  SRO/hotel: **aOR 0.49 (0.32, 0.73); p<0.001**  Friend: **aOR 0.38 (0.25, 0.57); p<0.001**  Shelter: **aOR 0.27 (0.13, 0.60); p<0.001**  Outdoors: **aOR 0.16 (0.09, 0.30); p<0.001** |
| Collazos et al 2009 [7] | 1352 people living with HIV from 69 hospitals (Spain) | **Educational** level | Undetectable viral load (baseline) – p=0.5  None or basic: 179 (21.0%)  Secondary: 62 (20.9%)  University: 27 (25.7%)  Undetectable viral load (12 months) – p=0.4  None or basic: 294 (59.2%)  Secondary: 101 (53.4%)  University: 39 (56.5%)  Mean (SD) viral load (log copies/mL) all patients (baseline) – p=0.4  None or basic: 3.93 (1.30)  Secondary: 3.83 (1.23)  University: 3.87 (1.34)  Median (IQR) CD4+ cell count (cells/μl) all patients (baseline) – **p=0.003**  None or basic: 249 (130 – 416.3)  Secondary: 280 (161 – 447)  University: 334 (182.5 – 492.5) | *Adjusted for gender, age, educational degree, transmission categories, prior antiretroviral experience, CD4 counts and viral load*  Baseline viral load  Educational level: p=0.8  Median CD4+ cell count all patient (baseline)  Educational level: p=0.0006 |
| D’Almeida et al 2016 [8] | 1246 people living with HIV who were treatment naïve at cART initiation and on cART for at least 12 months (France) | **Education** (ref: >2 year university degree)  **Employment** status (ref: employed)  **Occupational** grade (ref: executive)  **Material deprivation** | Sustained viral suppression or undetectable (VL<50 copies/mL) for at least 6 months  Elementary school: OR 0.6 (0.3, 1.0)  Middle school: OR 0.7 (0.4, 1.1)  High school: OR 0.7 (0.4, 1.3)  2 year university degree: OR 0.5 (0.3, 1.1)  Unemployed: **OR 0.5 (0.3, 0.8)**  On disability: OR 1.0 (0.5, 1.7)  Retiree: OR 1.3 (0.8, 2.3)  Inactive employment: OR 1.0 (0.5, 1.8)  Tradesperson: OR 0.6 (0.3, 1.4)  Associate professional or technician: OR 1.2 (0.7, 2.2)  Clerk: OR 0.7 (0.4, 1.2)  Manual worker: OR 0.8 (0.4, 1.3)  Other occupational grade: OR 0.8 (0.2, 3.9)  Material deprivation: **OR 0.6 (0.4, 0.8)** | *Adjusted for HIV-related (e.g. transmission subgroup, age at cART initiation), social factors (e.g. employment status, occupational grade etc.) health literacy and other health outcomes (e.g. hepatitis C coinfection)*  VL <50 copies/mL  Elementary school: **aOR 0.4 (0.2, 0.9)**  Middle school: aOR 0.6 (0.3, 1.1)  High school: aOR 0.7 (0.4, 1.4)  2 year university degree: aOR 0.5 (0.2, 1.1)  Unemployed: aOR 0.6 (0.3, 1.0)  On disability: aOR 0.9 (0.4, 1.8)  Retiree: aOR 0.8 (0.4, 1.7)  Inactive employment: aOR 1.0 (0.5, 2.2)  Tradesperson: aOR 0.7 (0.3, 1.6)  Associate professional or technician: aOR 1.4 (0.7, 2.8)  Clerk: aOR 1.1 (0.6, 2.0)  Manual worker: aOR 1.5 (0.8, 2.9)  Other occupational grade: aOR 1.1 (0.2, 6.6)  Material deprivation: aOR 1.0 (0.7, 1.9) |
| Del Amo et al 2017 [9] | 24,069 people living with HIV who are initiating cART (Austria, France, Germany, Greece, Italy, Spain, Switzerland and the Netherlands) | **Educational** level (ref: tertiary education) | Virological suppression (VL <400 copies/mL) – 1 year after cART initiation **(p<0.001)**  Incomplete primary education: **67%**  Primary education: **85%**  Secondary education: **82%**  Tertiary education: **87%**  Immunological response (change in square root CD4+ count after cART initiation) – baseline  Incomplete primary: **-1.24 (-1.50, -0.97)**  Primary: **-0.38 (-0.63, -0.12)**  Secondary: **-0.71 (-0.94, -0.48)**  CD4+ cell count at time 0 for baseline with tertiary education: **21.96 (21.49, 22.42); p<0.001**  Immunological response – in first 6 months  Incomplete primary: -0.14 (-0.57, 0.30)  Primary education: -0.41 (-0.83, 0.02)  Secondary education: 0.25 (-0.13, 0.64)  Annual rate of increase in first 6 months for baseline with tertiary education: **6.67 (6.37, 6.96); p<0.001**  Immunological response – after 6 months  Incomplete primary: 0.00 (-0.05, 0.06)  Primary education: -0.04 (-0.10, 0.02)  Secondary education: 0.05 (-0.01, 0.10)  Annual rate of increase after 6 months for tertiary education: **0.80 (0.76, 0.85); p<0.001** | *Adjusted for sex, age at cART initiation, year(s) of cART initation, transmission category, country of origin/birth, other clinical factors/virological response and stratified by cohort.*  VL <400 copies/mL **(p<0.001)**  Incomplete primary: **aHR 0.80 (0.76, 0.84)**  Primary: **aHR 0.93 (0.89, 0.97)**  Secondary: **aHR 0.97 (0.94, 1.01)** |
| Doshi et al 2017 [10] | 1,296,248 people living with HIV accessing medical care (USA) | Unstable/temporary **housing status** (ref: stable) |  | *Adjusted for age, race/ethnicity, and gender*  Viral suppression (VL <200 copies/mL) rates  Unstable housing: adjusted average annual change -0.3%  Temporary housing: adjusted average annual change **-0.5% (p<0.01)** |
| Dowshen et al 2016 [11] | 1,584 “behaviourally-infected youth” (including 66 young transgender women) of a multisite study (USA including Puerto Rico) | Unstable **housing status** | Detectable viral load  Unstable housing (young transgender women): 20% increased probability  Unstable housing (other behaviourally infected youth): 10% increase |  |
| Fadul et al 2017 [12] | 184 people living with HIV (USA) | **Education** (ref: high school grad)  Unstable **housing** status (ref: stable)  **Poverty level** [defined as % below or above federal poverty level (FPL). Categorised as 0-149% below FPL, 150% or more below FPL] | VL <200 copies/mL  <High school diploma: OR 0.58 (0.26, 1.29); p=0.181^‡^  Stable housing: OR 1.04 (0.239, 4.48); p=0.963^‡^  0-149% below FPL: OR 1.02 (0.51, 2.05); p=0.060^[[2]](#endnote-2)^ |  |
| Feldman et al 2015 [13] | 2896 men who have sex with men living with HIV enrolled in Ryan White Part A programs (USA) | **Education** (ref: ≥high school diploma)  Unstable/temporary **housing status** (ref: stable) | VL >200 copies/mL  <High school diploma: **OR 1.30 (1.09, 1.55); p<0.01**  Unstable housing: **OR 3.45 (2.74, 4,34); p<0.0001**  Temporary housing: **OR 1.85 (1.47, 2.31); p<0.0001**  CD4 <350 cells/mm^3^  <High school diploma: **OR 1.41 (1.18, 1.69); p<0.01**  Unstable housing: **OR 1.51 (1.21, 1.89); p<0.01**  Temporary housing: OR 1.06 (0.84, 1.34) | *Adjusted for age, race/ethnicity, primary language, education, country of birth, housing status, recent drug use, years living with HIV, ART status*  VL >200 copies/mL  <High school diploma: aOR 1.12 (0.91, 1.37)  Unstable housing: **aOR 2.33 (1.80, 3.02); p<0.0001**  Temporary housing: aOR 1.27 (0.98, 1.64) |
| Feller and Agins 2016 [14] | 11252 eligible adults living with HIV receiving treatment across 186 New York State HIV clinics (USA) | **Housing status** | VL (<200 copies/mL); % virally suppressed  Housing status (Stable)^[[3]](#endnote-3)^: **81% (p<0.001)**  Housing status (Supportive): **71% (p<0.001)**  Housing status (Unstable)^[[4]](#endnote-4)^: **60.6% (p<0.001)**  Housing status (Unknown)^[[5]](#endnote-5)^: **68.6% (p<0.001)** |  |
| Gardner et al 2015 [15] | 3,322 people receiving clinical care (Canada) | Personal **income** (ref: over $60,000)] |  | *Adjusted for all covariates shown (personal income, living alone, cigarette smoking, recent non-medicinal drug use in past 6 months, cannabis use) and year, age, sex, ethnicity, region, immigration, years living with HIV, time on ART, ART regimen, education, employment status, marital status, living with children, alcohol use, and clinic site*.  VL <200 copies/mL  Personal income <$20,000: aOR 0.97 (0.95, 0.99)  Personal income $20,000 to <$40,000: aOR 0.99 (0.97, 1.01)  Personal income $40,000 to <$60,000: aOR 1.00 (0.99, 1.02) |
| Gueler et al 2015 [16] | 2694 ART-naïve for virologic response at 6 months (Switzerland) | **Neighbourhood SEP** [categorised into 5 quintiles, where quintile 1: lowest SEP and quintile 5: highest SEP (ref: quintile 1)] |  | *Adjusted for age and sex*  Virological response to cART or viral suppression (VL <50 copies/mL) at 6 months  Neighbourhood SEP quintile 2: aOR 1.01 (0.78, 1.32)  Neighbourhood SEP quintile 3: aOR 1.13 (0.86, 1.48)  Neighbourhood SEP quintile 4: aOR 1.11 (0.85, 1.46)  Neighbourhood SEP quintile 5: **aOR 1.52 (1.14, 2.04)** |
| Haider et al 2019 [17] | 342 people living with HIV receiving HIV care (USA) | **Education** (ref: Bachelor’s or higher)  **Employment** (ref: unable to work/unemployed)  Annual household **income**  (ref: <$10,000) | VL <100 copies/mL  Grades 1-11: **OR 0.22 (0.06, 0.81); p=0.023**  Grade 12 or General Educational Diploma (GED): **OR 0.16 (0.05, 0.48); p=0.001**  Some College: **OR 0.29 (0.09, 0.89); p=0.031**  Full-time employment: **OR 2.21 (1.11, 4.4); p=0.025**  Part-time/other employment: OR 1.63 (0.76, 3.49)  $10,000-$24,999: OR 1.11 (0.57, 2.17); p=0.750  $25,000-$49,999: OR 1.78 (0.84, 3.80); p=0.135  ≥$50,000: **OR 6.17 (1.39, 27.35); p=0.016** | *Adjusted for age, sex, sexual orientation, race and other demographic and clinical characteristics*  VL <100 copies/mL  Grades 1-11: aOR 0.25 (0.04, 1.47); p=0.125  Grade 12 or GED: aOR 0.26 (0.06, 1.06); p=0.060  Some College: aOR 0.69 (0.18, 2.68); p=0.592  Full-time employment: **aOR 4.03 (1.27, 12.83); p=0.018**  Part-time/other employment: aOR 2.75 (0.78, 9.73); p=0.117  $10,000-$24,999: **aOR 0.21 (0.06, 0.73); p=0.014**  $25,000-$49,999: **aOR 0.11 (0.03, 0.52); p=0.005**  ≥$50,000: aOR 0.78 (0.06, 10.61); p=0.851 |
| Hussen et al 2018 [18] | 81 young Black gay, bisexual and other men who have sex with men living with HIV aged 18-24 years (USA) | **Education** (ref: completed high school/ GED/ post high school)  **Employment**  **Housing**: living with family or other (ref: lives alone)  Did not move in the last 6 months (ref: yes) | VL ≤ 40 copies/mL  Did not complete high school: OR 0.48 (0.19, 1.23); p=0.13  Working: **OR 2.41 (1.22, 4.77); p=0.011**  Housing – living with family: OR 0.90 (0.37, 2.18)  Housing – other: **OR 0.20 (0.09, 0.44); p=0.0002**  Did not move in last 6 months: **OR 2.8 (1.4, 5.6); p=0.0036** |  |
| Iralu et al 2010 [19] | 36 people living with HIV under Navajo AIDS Network case management (USA) | **Education** (ref: <high school education)  **Employment** (ref: unemployed including retired and “permanently/temporarily unable to work due to mental or physical disability)  Currently in permanent/stable **housing** (ref: not in permanent/stable housing)  Household **income** (ref: <$1,000 per month) | Viral load (log)  ≥High school education: t=0.12  Full-/part-time or occasional employment: t=1.89  Currently in permanent/stable housing: 3.63 (SD=2.61)  Not in permanent/stable housing: 5.59 (SD=3.92). t=1.64  ≥$1,000 per month: **t=2.20 (p<0.05)**  CD4 <200 cell/mm^3^  ≥High school education: t=1.46  Full-/part-time or occasional employment: t=0.75  Currently in permanent/stable housing: 432.3 (SD=164.5)  Not in permanent/stable housing: 357.5 (SD=233.8). t=1.04  ≥$1,000 per month: t=1.66 |  |
| Jansen et al 2009 [20] | 2045 people living with HIV (Germany) | **Income** | Viral load (<detection limit) – p=0.41  ≤€1,000: 67.7%  €1,001-2,500: 70.3%  >€2,500: 71.0% | *Adjusted for age, sex, viral load (<> detection limit), level of education*  CD4+ cell count (ref: <200/μl), where >€1,000 – **p<0.001**  200-499/μl: **aOR 1.803 (1.122, 2.899)**  ≥500/μl: **aOR 2.488 (1.542, 4.017)** |
| Kacanek et al 2019 [21] | 122 18-22-year-olds living with HIV (USA including Puerto Rico) | Annual household **income** (ref: >$40,000) | VL >400 copies/mL  ≤$10,000: **OR 5.17 (1.53, 17.51); p=0.01**  >$10,000-$40,000: OR 1.31 (0.49, 3.53); p=0.59 |  |
| Kalichman et al 2014 [22] | 364 men and 157 women living with HIV (USA) | **Food insecurity** (ref: food security) | Detectable viral load  Food insecurity: OR 1.44 (0.80, 2.61)  CD4 <500  Food insecurity: **OR 1.6 (1.04, 2.62); p<0.05** | *Adjusted for age, gender, poverty rate, reside in food desert, education, unstable housing, lack of transportation/clinic, lack of transportation/food etc.*  Detectable viral load  Food insecurity: aOR 1.88 (0.81, 4.41)  CD4 <500  Food insecurity: aOR 1.21 (0.64, 2.30) |
| Kalichman et al 2010 [23] | 344 men and women living with HIV/AIDS (USA) | **Food security** | Undetectable viral load  Food secure: **OR 1.7 (1.1, 3.0); p<0.05**  Most recent viral load, increased (ref: stayed the same)  Food insecure: **OR 2.2 (1.2, 4.2); p<0.01**  Most recent viral load, decreased (ref: stayed the same)  Food insecure: **OR 2.1 (1.1, 4.6); p<0.01**  Most recent T cells, increased (ref: stayed the same)  Food insecure: OR 0.6 (0.3, 1.1)  Most recent T cells, decreased (ref: stayed the same)  Food insecure: OR 1.3 (0.6, 2.9)  Most recent CD4 cell count  Food insecurity: **t=2.2 (p<0.05)** |  |
| Lacombe-Duncan et al 2019 [24] | 50 transwomen living with HIV (Canada) | < high school **education**  Insecure **housing** [defined as self-contained room, transition house, halfway house, safe house, couch surfing, outdoors on street, parks, or in a car. Reference: secure - apartment (own/rent) or a house (own/rent)]  <$20,000 personal **income** | VL <50 copies/mL  <High school education: Proportion difference -5.7 (-51.7, 20.9)  Insecure housing: Proportion difference: 12.1 (-13.8, 57.7)  <$20,000: Proportion difference: -10 (- 25.6, 39.4) |  |
| Lim et al 2015 [25] | 1,698 New York City people living with HIV/AIDS with both jail incarceration and homelessness (USA) | Temporary, continuous incarceration or continuing **shelter use** (ref: decreasing shelter use) | VL <400 copies/mL  Temporary (intermittent jail incarceration and shelter stays between January 2001–May 2005): **PR 0.67 (0.50, 0.90)**  Continuous incarceration (extensive amounts of uninterrupted time in jail from January 2001– May 2005): **PR 0.62 (0.43, 0.88)**  Continuing shelter use (extensive amounts of time in shelters (average 728 days) without much interruption): PR 0.71 (0.52, 1.22) |  |
| Ludema et al 2016 [26] | 1481 women living with HIV (USA) | Annual **income** (ref: <$6,000) | VL >200 copies/mL  $6001-$12,000 per year: HR 0.84 (0.68, 1.03)  $12,001-$18,000 per year: HR 0.77 (0.59, 1.00)  >$18,000 per year: **HR 0.70 (0.55, 0.90)** | *Adjusted for (a) study site, age, race, and lowest CD4 (at baseline), and average log10 (viral load) in the 3 visits before index date (spline) or (b) study site, age, race, and lowest CD4 (at baseline).*  VL >200 copies/mL  $6001-$12,000 per year: aHR 1.09 (0.84, 1.41). aHR 0.93 (0.73, 1.18)  $12,001-$18,000 per year: aHR 1.08 (0.80, 1.46). aHR 0.88 (0.66, 1.17)  >$18,000 per year: aHR 1.06 (0.80, 1.41). aHR 0.79 (0.60, 1.02) |
| Marshall et al 2016 [27] | 706 people living with HIV who use drugs who were community-recruited (Canada) | **Educational** attainment (ref: <high school)  **Employment**  **Homeless** (yes vs. no) | Undetectable viral load or viral suppression (VL <50 copies/mL)  ≥High school completion: PR 1.19 (0.99, 1.44); p=0.071  Any formal employment: PR 1.07 (0.84, 1.35); p=0.597  Homeless**: PR 0.49 (0.37, 0.64); p<0.001** | *Adjusted for sociodemographics (age, sex at birth, race/ethnicity etc.), substance use, addiction treatment, (educational attainment) and other confounders (e.g. year of interview).*  Undetectable viral load or viral suppression (VL <50 copies/mL)  ≥High school completion: aPR 1.19 (0.99, 1.42); p=0.058  Any formal employment: aPR 1.02 (0.81, 1.27); p=0.896  Homeless**: aPR 0.55 (0.42, 0.71); p<0.001** |
| Miller et al 2006 [28] | 892 participants in analysis who were entered to the HIV/AIDS Drug Treatment Program of the British Columbia Centre for Excellence in HIV/AIDS (Canada) | Stable **housing**  **Income** <$10,000 CAN (ref: no) | VL <500 copies/mL  Stable housing: **RR 1.56 (1.23, 1.97)**  <$10,000 CAN: **RR 0.66 (0.55, 0.77)** |  |
| Mimiaga et al 2019 [29] | 296 adult residents of Rhode Island living with HIV (USA) | **Education** (ref: some College or more)  **Unemployed**(ref: employed)  **Homeless** in the past 12 months  Annual household **income** (ref: yes, income)  **Financial** concerns in the past 12 months (ref: no) | Virological non-suppression  <High school: OR 1.93 (0.70, 5.37); p=0.21  High school degree or equivalent: OR 0.92 (0.25, 3.43); p=0.90  Unemployed: OR 0.78 (0.30, 2.02); p=0.61  Homelessness: **OR 3.47 (1.12, 10.76); p=0.03**  No income: **OR 3.30 (1.07, 10.18); p=0.04**  Financial concerns: OR 2.12 (0.84, 5.37); p=0.11 |  |
| Moore et al 2016 [30] | 719 men who have sex with men (Canada) | **Education** (student status)  **Income** <$15,000 | VL ≥200 copies/mL  Current student: OR 1.30 (0.42, 4.08)  <$15,000: **OR 5.85 (2.35, 14.54)** | *Adjusted for age, ethnicity, sexual orientation, born in Canada and other social and behavioural characteristics*  VL ≥200 copies/mL  <$15,000: **aOR** **6.43 (2.08, 19.89)** |
| Oliver et al 2019 [31] | 248 women living with HIV with ≥1 prenatal visit (USA) | **Education** (ref: ≥GED or high school) | Poor viral suppression (VL ≥200 copies/mL)  <12^th^ grade: **RR 1.17 (1.08, 1.27)**  Unknown: RR 1.32 (0.98, 1.78) | *Adjusted for age, race/ethnicity, HIV related factors, health behaviours and other social factors*  Poor viral suppression (VL ≥200 copies/mL)  <12^th^ grade: aRR 1.09 (0.96, 1.24)  Unknown: **aRR 1.31 (1.07, 1.60)** |
| Persson et al 1994 [32] | 47 men who have sex with men (Sweden) | **Social class** (ref: social class II – middle range civil servants/employees) | Low CD4 cell count (< median)  Social class III (skilled/unskilled workers): OR 1.5 (0.5, 4.9) |  |
| Raho-Moussa et al 2019 [33] | 475 people living with HIV treated with ART for at least 6 months (France) | **Educational** level  **Employment**  **Deprivation** [defined using Evaluation of Deprivation and Inequalities in Health Examination Centres (EPICES) or using individual components of EPICES score (including **financial difficulties** in previous month, owner of his/her **housing**)] | Detectable viral load (VL >50 copies/mL)  <Secondary school: OR 0.88 (0.50, 1.54); p=0.66^‡^  Formal, informal, full-/part-time employment: OR 0.74 (0.44, 1.26); p=0.267^‡^ | *Adjusted for reporting ART tiredness, younger age, and a previous AIDS event (and if using individual components of EPICES owner of housing)*  VL >50 copies/mL  Deprivation (owner of his/her housing): **aOR** **8.96 (1.19, 67.46)**  Deprivation (EPICES score ≥30.17): **aOR 2.89 (1.18, 7.04)**  Deprivation defined using individual components of EPICES score (Financial difficulties in previous month): **aOR 2.90 (1.38, 6.10)** |
| Rebeiro et al 2018 [34] | 2541 adults in viral suppression analysis (USA) | Neighbourhood **socioeconomic context** risk score (ref: quintile 1 – least adverse) | VL <200 copies/mL among those with ≥1 clinic visit  Neighbourhood socioeconomic context risk score 2^nd^ quartile: RR 0.93 (0.85, 1.02)  Neighbourhood socioeconomic context risk score 3^rd^ quartile: RR 0.96 (0.87, 1.06)  Neighbourhood socioeconomic context risk score 4^th^ quartile - most adverse: RR 0.87 (0.79, 0.97) | *Adjusted for individual year of birth, sex, race/ethnicity, and time since enrolment in HIV care*  VL <200 copies/mL among those with ≥1 clinic visit  Neighbourhood socioeconomic context risk score 2^nd^ quartile: ARR 0.94 (0.87, 1.03).  Neighbourhood socioeconomic context risk score 3^rd^ quartile: ARR 0.97 (0.88, 1.06).  Neighbourhood socioeconomic context risk score 4^th^ quartile - most adverse: ARR 0.88 (0.80, 0.97) |
| Robinson and Knowlton 2016 [35] | 383 disadvantaged people living with HIV (formerly and/or currently inject drugs) (USA) | **Education** (ref: ≤8^th^ grade) | VL ≤40 copies/mL  Some high school – men: IRR 1.37 (0.74, 2.53)  High school/GED – men: IRR 1.54 (0.84, 2.82)  Some College/above – men: IRR 1.57 (0.85, 2.92)  Some high school – women: IRR 1.72 (0.69, 4.30)  High school/GED – women: IRR 1.74 (0.69, 4.37)  Some College/above – women: IRR 2.25 (0.90, 5.63); p<0.10 | *Adjusted for substance use, mental illness, physical function, familial conflict, social support and other intermediary factors*  VL ≤40 copies/mL  Some high school – men: aIR 1.64 (0.82, 3.29)  High school/GED – men: aIR 1.75 (0.88, 3.48)  Some College/above – men: aIR 1.89 (0.94, 3.79); p<0.10  Some high school – women: aIR 2.05 (0.80, 5.30)  High school/GED – women: aIR 2.03 (0.76, 5.39)  Some College/above – women: **aIR 2.60 (1.00, 6.77); p<0.05** |
| Santos et al 2014 [36] | 314 transwomen (USA) | Homeless or marginalised **housing** (ref: stable) |  | *Adjusted for age, race/ethnicity, history of injection drug use, health insurance*  VL ≤200 copies/mL  Homeless or marginally housed: **aOR** **0.05 (0.01, 0.51); p=0.011** |
| Saracino et al 2018 [37] | 8,023 people living with HIV (Italy) | **Education** (ref: elementary school)  **Employment** (ref: full-time worker) |  | *Adjusted for CD4 count, viral load, pregnancy status, smoking*  VL <50 copies/mL  Junior high school: aHR 1.10 (0.95, 1.26); p=0.214  High school: **aHR 1.16 (1.01, 1.34); p=0.041**  University: **aHR 1.21 (1.03, 1.43); p=0.020**  Missing data (education): aHR 1.08 (0.93, 1.25); p=0.326  Unemployed: **aHR 0.87 (0.79, 0.96); p=0.006**  Self-employed: aHR 0.97 (0.89, 1.05); p=0.406  Temporary employed: aHR 0.84 (0.69, 1.02); p=0.073  Student: aHR 0.93 (0.79, 1.10); p=0.392  Retired: aHR 1.01 (0.85, 1.19); p=0.914  Housewife: aHR 0.88 (0.73, 1.05); p=0.163  Other/unknown employment: aHR 0.91 (0.76, 1.09); p=0.299  Missing data (employment): aHR 0.96 (0.88, 1.06); p=0.453 |
| Sayles et al 2012 [38] | 11,397 people living with HIV in the Ryan White system, a publicly funded system of care for people living with HIV in Los Angeles County who are uninsured (USA) | Non-permanent **housing/homeless** living situation (ref: permanent)  **Income** by federal poverty level, FPL (ref: >100 FPL) | VL >200 copies/mL  Non-permanent housing/homeless: **OR 1.72 (1.53, 1.92); p<0.001**  ≤100% FPL: **OR 1.56 (1.43, 1.71)** | *Adjusted for gender, race/ethnicity, age, income, insurance status, mode of exposure, substance use, incarceration, and clinical variables (HIV diagnoses < 1 year ago, CD4 count, currently on ART)*  VL >200 copies/mL  Non-permanent housing/homeless: aOR 1.05 (0.91, 1.21)  ≤100% FPL: **aOR** **1.27 (1.15, 1.41)** |
| Schafer et al 2012 [39] | 251 participants from the UVa Ryan White clinic (RWC) (USA) | **Education** (ref: completed 12^th^ grade)  **Socioeconomic status** [defined by payscale, co-pay for patient. Categorised as no-copay (low SES), 5-70% co-pay (mid-range SES), 100% co-pay (high SES)] | Detectable HIV viral load  Did not complete 12^th^ grade: OR 1.12 (0.57, 2.19); p=0.745  Payscale: p=0.066  CD4 <200  Did not complete 12^th^ grade: OR 1.19 (0.33, 4.23); p=0.787  Payscale: p=0.114 | *Adjusted for age, race, behavioural factors*  Detectable HIV viral load  5-70% co-pay for patient: **aRR** **0.39 (0.16, 0.94); p=0.035**  CD4 <200  5-70% co-pay for patient: Not significant |
| Shacham et al 2010 [40] | 514 individuals presenting at an urban U.S. HIV clinic (USA) | **Education** (ref: >High school)  **Employment** status (ref: employed)  Considers self **homeless** | VL ≥400 copies/mL  ≤High school diploma: **OR 3.23 (1.58, 6.58)**  Unemployed/disability benefits: **OR 1.85 (1.11, 3.85)**  Homeless: **OR 2.42 (1.45, 8.46)** | *Adjusted for gender, race, age, employment status, considers self homeless, depressive disorders, HAART type and drug regimen*  VL ≥400 copies/mL  ≤High school diploma: **aOR 2.32 (1.08, 5.00)** |
| Shacham et al 2013 [41] | 762 individuals at HIV Clinic (USA) | % ≥median **unemployed** (ref: <median)  % ≥median below **poverty** line (ref: <median) | VL <400 copies/mL  % unemployed: OR 0.91 (0.66, 1.25)  % below poverty line: OR 0.91 (0.66, 1.25)  CD4 <200 cells/μl  % unemployed: **OR 0.66 (0.45, 0.96)**  % below poverty line: **OR 0.62 (0.43, 0.90)** | *Adjusted for variables that were significant in unadjusted models: age, gender, race, education, income, depression (except depression outcome), number of sex partners and unprotected sex events*  VL <400 copies/mL  % unemployed: aOR 0.84 (0.59, 1.21)  % below poverty line: aOR 0.78 (0.55, 1.12)  CD4 <200 cells/μl  % unemployed: aOR 0.73 (0.47, 1.13)  % below poverty line: **aOR 1.56 (1.05, 2.44)** |
| Sobrino-Vegas et al 2012 [42] | 4549 adults with confirmed HIV (Spain) | **Educational** level (ref: low – no education or with primary education) | Virological response (VL<50 copies/mL) – 6 months  Medium – completed secondary: **OR 1.46 (1.16, 1.84)**  High – completed university: **OR 1.98 (1.46, 2.68)**  Virological response (VL<50 copies/mL) – 1 year  Medium – completed secondary: **OR 1.52 (1.20, 1.92)**  High – completed university: **OR 1.88 (1.44, 2.45)**  Immunological response to treatment (increase in CD4+ ≥50 cells/mm^3^) – 6 months  Medium – completed secondary: **OR 1.45 (1.17, 1.80)**  High – completed university: **OR 1.78 (1.31, 2.42)**  Immunological response to treatment (increase in CD4+ ≥50 cells/mm^3^) – 1 year  Medium – completed secondary: **OR 1.45 (1.09, 1.92)**  High – completed university: **OR 2.37 (1.69, 3.32)** | *Adjusted for sex, age at treatment, transmission category, CD4+ T-cell count and viral load before initiating treatment*  Virological response (VL<50 copies/mL) – 6 months  Medium – completed secondary: **aOR 1.41 (1.10, 1.80)**  High – completed university: **aOR 1.80 (1.22, 2.65)**  Virological response (VL<50 copies/mL) – 1 year  Medium – completed secondary: aOR 1.27 (1.01, 1.61)  High – completed university: aOR 1.33 (0.97, 1.83)  Immunological response to treatment (increase in CD4+ ≥50 cells/mm^3^) – 6 months  Medium – completed secondary: aOR 1.26 (1.01, 1.57)  High – completed university: aOR 1.41 (1.00, 2.00)  Immunological response to treatment (increase in CD4+ ≥50 cells/mm^3^) – 1 year  Medium – completed secondary: aOR 1.24 (0.93, 1.66)  High – completed university: **aOR 1.75 (1.22, 2.51)** |
| Tymejczyk et al 2018 [43] | 1045 persons (SHC electronic medical records matched with longitudinal data from NYC HIV Surveillance Registry) (USA) | Neighbourhood **poverty** level, NPL (ref: <10%) | VL ≤200 copies/mL  10-20% NPL: OR 0.67 (0.41, 1.10)  >20-30% NPL: OR 0.92 (0.58, 1.47)  >30% NPL: **OR 0.53 (0.32, 0.86); p<0.01**  Unknown NPL (metropolitan NYC): **OR 0.33 (0.14, 0.77); p<0.01** | *Adjusted for sex assigned at birth, age, race/ethnicity, residence, primary language, transmission risk, sexual health and HIV care*  VL ≤200 copies/mL  10-20% NPL: aOR 0.66 (0.38, 1.15)  >20-30% NPL: aOR 1.00 (0.59, 1.70)  >30% NPL: **aOR 0.51 (0.29, 0.89); p<0.05**  Unknown NPL (metropolitan NYC): aOR 0.60 (0.24, 1.52) |
| Wagoner et al 2016 [44] | 382 people living with HIV with a HIV viral load available 12 months ± 90 days from the time of entry into HIV care (USA) | **Education** (ref: ≤diploma/GED) |  | *Adjusted for age, race, sex/sexual behaviour, living arrangement, church attendance, HIV related factors (e.g. CD4 count) and other health status factors*  VL ≥200 copies/mL  Some College or more: **aOR 0.5 (0.2, 0.9); p<0.05** |
| Weiser et al 2013 [45] | 284 homeless and marginally housed individuals living with HIV (USA) | <High school **education**  Any **food** **insecurity** [including mild, moderate, or severe food insecurity (ref: food secure)]  Recent **homelessness** [defined as living in shelter or on street in past 90 days]  **Income** <median ($916) | VL >100 copies/mL  <High school: OR 1.14 (0.74, 1.76)  Any food insecurity: **OR 1.36 (1.1, 1.68); p<0.01**  Recent homelessness: **OR 1.98 (1.25, 3.14); p<0.01**  Income < median: OR 0.99 (0.68, 1.46)  CD4 <200 cells/mm³  <High school: OR 1.02 (0.61, 1.70)  Any food insecurity: **OR 1.22 (1.04, 1.42); p<0.05**  Recent homelessness: **OR 1.42 (1.07, 1.88); p<0.05**  Income < median: OR 1.07 (0.67, 1.72) | *Adjusted for sociodemographic (e.g. age, sex, ethnicity, education etc.) and clinical variables.*  VL >100 copies/mL  Any food insecurity: **aOR 1.29 (1.04, 1.61); p<0.05**  Recent homelessness: **aOR** **1.89 (1.16, 3.07); p<0.05**  CD4 <200 cells/mm³  Any food insecurity: **aOR 1.26 (1.01, 1.56); p<0.05**  Recent homelessness: **aOR 1.65 (1.09, 2.49); p<0.05** |
| Whelan et al 2019 [46] | 549 individuals living with HIV (USA) | **Housing** instability – homeless or unstably housed (ref: permanent/stable) | VL ≥200 copies/mL  Housing instability: **RR 1.19 (1.05, 1.35)** |  |
| Wilson et al 2018 [47] | 159 transwomen of colour living with HIV (USA) | **Food insecurity** in the last 6 months (ref: never)  Unstable **housing** in last 6 months (ref: never)  **Income** source in the last 6 months [categorised as illicit (drug dealing/selling, sex work, panhandling, or boosting/stealing) or licit income sources (employment, income from partner, or income from family) or entitlements (Food Stamps, General Assistance (GA), Aid to Families with Dependent Children (AFDC), Social Security, Supplemental Security Income/Social Security Disability Insurance (SSI/SSDI), Unemployment)] (ref: only licit sources) |  | *Adjusted for site, age, race/ethnicity, education level, and marital status; did not adjust for income since all participants were very low income.*  Undetectable viral load in past 6 months  Food insecure rarely/sometimes: aPR 0.95 (0.67, 1.33)  Food insecure most of the time/always: aPR 1.15 (0.68, 1.92)  Unstable housing rarely/sometimes: aPR 1.07 (0.71, 1.63)  Unstable housing often: aPR 0.78 (0.43, 1.41)  Any illicit income source: aPR 0.79 (0.49, 1.25)  CD4 <500 in past 6 months  Food insecure rarely/sometimes: aPR 1.42 (0.87, 2.32)  Food insecure most of the time/always: aPR 0.74 (0.29, 1.88)  Unstable housing rarely/sometimes: aPR 1.88 (0.93, 3.8)  Unstable housing often: **aPR 1.84 (1.05, 3.26)**  Any illicit income source: aPR 1.05 (0.63, 1.73) |
| Yehia et al 2014 [48] | 12,759 adults living with HIV using multiple clinics for primary HIV care (Ryan White Program funded) (USA) | **Income** (ref: <$10,000) |  | *Adjusted for age, sex, race/ethnicity and other demographic and clinical characteristics*  VL ≤200 copies/mL  $10,000-$19,999: AOR 1.08 (0.98, 1.18)  $20,000-$49,999: **AOR 1.34 (1.15, 1.55)**  ≥$50,000: AOR 1.40 (1.01, 1.93) |

**Outcome 2: Medication (non-) adherence**

| **Author** | **Population (country)** | **Measure of social determinant** | **Main unadjusted results estimate (95% CI)*** | **Main adjusted results estimate (95% CI)*** |
| --- | --- | --- | --- | --- |
| Abgrall et al 2014 [49] | 200 native Sub-Saharan African individuals living in France adherent to cART at enrolment (France) | **Housing** status (living with others, unstable; ref: homeowner/renter) |  | *Adjusted for marital status, HIV status disclosure and ART effectiveness perception*  cART adherence failure (decrease in adherence rate from 80-100% to <80%)  Living with others/unstable housing status: **aOR 3.30 (1.04, 10.50); p=0.04** |
| Almeida-Brasil et al 2018 [1] | 566 adults living with HIV and hepatitis C receiving cART treatment (Canada) | **Education** (ref: ≤high school education)  **Employment** status (ref: not working)  Income-related **food insecurity** (defined as Household Food Security Survey Module, HFSSM, score ≥2  Gross monthly **income** $1000CAD or less | cART non-adherence (“having missed at least one dose over the past 4 days”)  More than high school education (i.e. College or university): OR 0.97 (0.64, 1.46)  Working: OR 1.12 (0.73, 1.73) | *Adjusted for age, sex, education, housing status, employment status, income etc.*  cART non-adherence  ≤$1000CAD: aOR 1.04 (0.74, 1.45)  Income-related food insecurity: **aOR 1.77 (1.26, 2.48); p<0.01** |
| Berg et al 2004 [50] | 113 people who currently/formerly used opioid and are living with HIV (USA) | Long-term **housing** (defined as more than 3 years living in current residence) |  | *Adjusted for gender and intermediary determinants (e.g. substance/alcohol use, depression etc.)*  ART adherence rate (divided number of actual MEMS caps openings by number of prescribed doses)  Long-term housing: **β** **(16) [5.3, 26.7]; p=0.004** |
| Burch 2018 [5] | 2704 individuals living with HIV recruited from eight HIV outpatient clinics (UK) | University **education**  **Employment**  **Housing** status – unstable or renting (ref: homeowner)  Enough **money** for basic needs? (financial hardship) (ref: always) | Self-reported ART non-adherence (≥2 consecutive missed days of ART in the past 3 months or ≥1 missed dose in the last 2 weeks)  No university education: **PR 1.18 (1.05, 1.33); p=0.0041**  Unemployed: **PR 1.24 (1.11, 1.38); p=0.0002**  Renting: **PR 1.44 (1.27, 1.65); p<0.0001**  Unstable housing: **PR 1.76 (1.47, 2.10); p<0.0001**  Enough money for basic needs? Mostly: **PR 1.44 (1.24, 1.66)**  Enough money for basic needs? Sometimes: **PR 1.88 (1.62, 2.17)**  Enough money for basic needs? No: **PR 1.82 (1.55, 2.14)** | *Adjusted for gender/sexual orientation and age*  Self-reported ART non-adherence  No university education: **aPR 1.18 (1.05, 1.32); p=0.0050**  Unemployed: **aPR 1.29 (1.16, 1.45); p<0.0001**  Renting: **aPR 1.36 (1.19, 1.56); p<0.0001**  Unstable housing: **aPR 1.62 (1.35, 1.95); p<0.0001**  Enough money for basic needs? Mostly: **aPR 1.43 (1.24, 1.65)**  Enough money for basic needs? Sometimes: **aPR 1.85 (1.59, 2.15)**  Enough money for basic needs? No: **aPR 1.78 (1.50, 2.11)** |
| Carrieri et al 2003 [51] | 96 people who inject drugs living with HIV (30 women, 66 men) initially adherent to HAART in hospital clinics (France) | **Educational** level (ref: <high school certificate)  **Employment**  Comfortable **housing** | Failure to maintain HAART adherence (<80%) during 18-months follow up  High school certificate: OR 0.60 (0.20, 1.80); p=0.347  Has a regular job: OR 1.5 (0.6, 3.8); p=0.362  Comfortable housing: OR 0.7 (0.30, 2.10); p=0.555 |  |
| Chitsaz et al 2013 [52] | 1166 adults aged 18 or older who knew HIV diagnosis before being jailed (USA) | **Education**  Paid **work** in 30 days pre-incarceration  **Food insecurity**  **Homelessness** | ≥95% ART adherence among subjects who received ART in 7 days prior to incarceration  Education: p<0.10  Paid work: **OR 2.03 (1.27, 3.24); p=0.003**  Food insecurity: **OR 0.68 (0.46, 0.98); p=0.039**  Homelessness: **OR 0.61 (0.42, 0.87); p=0.007** | *Adjusted for gender, age race/ethnicity, food insecurity, education and intermediary determinants (e.g. health insurance status)*  ≥95% ART adherence  Paid work: **aOR 2.02 (1.21, 3.36); p=0.007**  Food insecurity: aOR 0.97 (0.62, 1.54); p=0.908  Homelessness: aOR 0.72 (0.47, 1.10); p=0.129 |
| Collazos et al 2009 [7] | 1352 people living with HIV from 69 hospitals (Spain) | Degrees of **education** | HAART adherence rates  None or basic (ref: secondary): **OR 0.62 (0.45, 0.84); p=0.002**  None or basic (ref: university): OR 0.68 (0.40, 1.14); p=0.10 |  |
| Creasy et al 2019 [53] | 5143 individuals which currently identified as male and as Black/African American recruited from Black Pride events (USA) | **Homeless** in the past 12 months [defined as living on the street, in shelter, in SRO or care] | Days/week missed dose (4-7 times/week, 2-3 times/week, once/week, <once/week, never)  Homeless: W or χ²: 7792.0 (p=0.282)  Last missed dose (“never,” “over 3 months ago,” “1–3 months ago,” “3–4 weeks ago,” “1–2 weeks ago,” or “within the week")  Homeless: **W or χ²: 38113.0 (p<0.001)** |  |
| Dorz et al 2003 [54] | 109 people living with HIV: 88 male, 21 female adults (Italy) | **Education** (ref: >8 years)  **Employment**  **Economic** difficulties | Self-reported non-adherence (<80%) of prescribed therapy in past week  ≤8 years education: χ² / F: 2.672 (not significant)  Has a job: χ² / F: 0.422 (not significant)  Economic difficulties: χ²/F= 5.62 (not significant) |  |
| Gebo et al 2003 [55] | 196 people living with HIV taking at least 1 antiretroviral medication, awaiting a visit with their primary care provider in an urban hospital clinic) (USA) | **Material deprivation** (defined as eating <2 meals/day; running out of money in past 90 days) | <90% ART adherence  Eating <2 meals: **OR 4.01 (1.44, 11.14); p=0.008**  Running out of money in past 90 days: **OR 2.21 (1.16, 4.21); p=0.016** [not included in multivariate due to co-linearity] | <90% ART adherence  *Adjusted for drug use, binge drinking, social pressures, beliefs etc.*  Eating <2 meals: **aOR** **3.31 (1.11, 9.92); p=0.032**  ART non-adherence (risk factor of IDU)  *Adjusted for sexual contact, drug use, social pressures (risk factor other than IDU)*  Eating <2 meals: **aOR** **17.54 (1.92, 160.4); p=0.011**  <90% ART adherence (women)  *Adjusted for men, drug use, social pressure*  Eating <2 meals: **aOR** **8.32 (1.31, 52.8); p=0.025** |
| Golin et al 2002 [56] | 117 people living with HIV with adherence measured for at least 2 four-week periods at a county hospital HIV clinic (USA) | **Education**, % (ref: other)  **Employment**, %  **Income** ≤$10,000 per year (ref: >$10,000/y) | ART adherence [protease inhibitor (PI), non-nucleoside reverse transcriptase inhibitor (NNRTI) or highly active antiretroviral therapy (HAART)]  <high school graduate: 0.659  High school graduate: 0.739  College graduate: 0.750 (p=0.06)  % working (yes): 0.722  % working (no); 0.709; p=0.71 | *Adjusted for age, ethnicity, (income level), alcohol/drug use, dose frequency and number of reminders, receive ART and other social, behavioural, clinical characteristics*  ART adherence  High school education: **parameter estimate =0.058 (SE = 0.029); p=0.05**  ≤$10,000/y: **parameter estimate =0.066 (SE = 0.030); p=0.03** |
| Gordillo et al 1999 [57] | 366 people living with HIV on ART treatment (Spain) | **Education** (ref: no studies)  **Employment** (ref: pensioner) | ART adherence ('good': >90%)  Primary studies: OR 2.15 (0.62, 7.50)  Secondary school: **OR 3.69 (1.03, 13.20)**  Vocational training: OR 2.08 (0.55, 7.83)  University studies: **OR 4.0 (1.10, 14.50)**  Working: **OR 2.24 (1.35, 3.71)**  Unemployed: OR 1.13 (0.60, 2.11)  Other work situation: OR 1.54 (0.64. 3.69)^[[6]](#endnote-6)^ ^‡‡^ |  |
| Johnson et al 2003 [58] | 2765 adults living with HIV taking ART (USA) | **Educational** achievement [categorised as <high school, high school, some College, College Graduate]  **Employment** status  **Homeless**/**shelter** in the past year  Ever lived in shelter/welfare **housing** | ART non-adherence (<90%) in past 3 days  Lower educational achievement: **χ²=7.89 (3); p<0.05**  Working: χ²=0.48 (1)  Homeless/shelter in past year: **OR 1.38 (1.02, 1.85); p=0.035**  Ever lived in shelter/welfare housing: **χ²=19.67 (1); p<0.001**  ART adherence (≥90%)  Homeless in past year: **χ²=16.89 (1); p<0.001** |  |
| Kacanek et al 2019 [21] | 122 18-22-year-olds living with HIV (USA including Puerto Rico) | Annual household **income** (ref: >$40,000) | Self-reported ART non- adherence (“youth or caregiver report of one or more missed ART doses in the past week, associated with unsuppressed viral load in previous week”)  ≤$10,000: OR 2.94 (0.97, 8.89); p=0.06  >$10,000-$40,000: OR 2.17 (0.92, 5.12); p=0.08 | *Adjusted for race and other social demographics (e.g. caregiver marital status)*  Self-reported ART non- adherence  ≤$10,000: aOR 2.41 (0.79, 7.39); p=0.12  >$10,000-$40,000: aOR 1.96 (0.82, 4.66); p=0.13  *Data extracted of 18-22 year olds at time of adherence evaluation.* |
| Kalichman and Grebler 2010 [59] | 188 men and women living with HIV/AIDS who demonstrated poor health literacy (USA) | Years of **education**  **Employment**  **Income** (ref ≥$10,000)  **Poverty-related experience**  Sum of **poverty-related stressors**  **Poverty-related stress** | ≥85% ART adherence  Years of education: OR 1.00 (0.85, 1.18)  Unemployed: OR 0.87 (0.63, 1.19)  Income <$10,000: OR 1.01 (0.66, 1.56)  Worried about having a place to stay: OR 0.56 (0.30, 1.06)  Had to choose between paying for food and medications: **OR 0.34 (0.15, 0.78)**  Ran out of food and had no money for more: **OR 0.29 (0.15, 0.56)**  Worried about food running out: **OR 0.26 (0.13, 0.49)**  Cut down quantities of skipped meals to conserver food: **OR 0.33 (0.14, 0.72)**  Lacked food and ate less than needed: **OR 0.29 (0.13, 0.65)**  Lacked food and was hungry: **OR 0.35 (0.14, 0.90); p<0.05**  Lacked food and did not eat for a whole day: OR 0.66 (0.26, 2.30)  Sum of poverty-related stressors: **OR 0.73 (0.62, 0.86); p<0.01** | *Adjusted for social stressors, depression, internalised AIDS stigma, drug use*  85% ART adherence  Poverty-related stress: **aOR 0.72 (0.59, 0.89); p<0.01**  75% ART adherence  Poverty-related stress: **aOR 0.74 (0.62, 0.88); p<0.01** |
| Kalichman et al 2010 [23] | 344 men and women living with HIV/AIDS (USA) | Years of **education**  **Employment**  **Food security**  **Food insufficiency** (defined using an adapted version of the US Food Security Scale)  Unstable **housing** (homeless or living in a shelter)  **Income** <$1000 | Self-report adherence rating  Food secure: **t=3.2 (p<0.01)**  Unannounced pill count adherence  Food secure: **t=2.7 (p<0.01)** | *Adjusted for education, unemployment, income <$1000, food insufficiency and several intermediary factors (depression, social support, alcohol/drug use)*  80% ART adherence  Years of education: aOR 0.9 (0.7, 1.2)  Unemployment: aOR 1.1 (0.3, 3.5)  Food insufficiency: **aOR 0.3 (0.1, 0.8); p<0.05**  Unstable housing: aOR 0.6 (0.3, 1.8)  <$1000: aOR 0.9 (0.3, 2.6)  90% ART adherence  Years of education: aOR 0.9 (0.7, 1.2)  Unemployment: aOR 2.3 (0.9, 6.4)  Food insufficiency: **aOR 0.3 (0.1, 0.9); p<0.05**  Unstable housing: aOR 1.8 (0.7, 4.2)  <$1000: aOR 0.8 (0.3, 2.0) |
| Kalichman et al 2014 [22] | 364 men and 157 women living with HIV (USA) | **Food insecurity** | 85% ART adherence  Food insecurity: **OR 0.5 (0.31, 0.75); p<0.01** | *Adjusted for age, gender, poverty rate, reside in food desert, education, unstable housing, lack of transportation/clinic, lack of transportation/food etc*  85% ART adherence  Food insecurity: **aOR** **0.43 (0.2, 0.92); p<0.05** |
| Keith McInnes et al 2013 [60] | 1871 people living with HIV (USA) | **Education** (ref: high school or less)  **Employment**  Ever **homeless**  Annual household **income** (ref: <$6,000) |  | *Adjusted for age, sex, race/ethnicity and other sociodemographic characteristics and health behaviours (patient electronic personal health record users vs. all other categories)*  ART adherence (1-MEDOUT ≥0.90)  Some College or technical education: aOR 1.00 (0.81, 1.22)  College graduate or higher: aOR 1.05 (0.72, 1.54)  Employed for wages: aOR 0.96 (0.80, 1.14)  Ever homeless: OR 0.95 (0.81, 1.11)  $6,000-11,999: aOR 1.12 (0.89, 1.42)  $12,000-24,999: aOR 0.94 (0.71, 1.26)  $25,000-$49,999: **aOR 1.51 (1.06, 2.16)**  ≥$50,000: aOR 0.76 (0.43, 1.36) |
| Kleeberger et al 2004 [61] | 597 men living with HIV reporting use of HAART and provided adherence data (USA) | **Education**  **Income** | Negative change (%): visit-pairs with 100% adherence  College or more: 562 visit pairs with 100% adherence (9% negative change)  Less than College: 372 (14%) **p=0.03**  <$50,000: -12%  ≥50,000: -10%  Positive change (%): visit-pairs with <100% adherence  College or more: 101 (69%)  Less than College: 85 (74%) p=0.51  <$50,000: 71%  ≥50,000: 74% |  |
| Koehn et al 2020 [62] | 99 people living with HIV who completed a 12-month follow-up interview (Canada) | **Educational** level (ref: some post-secondary and above)  **Employment**  **Food insecure** (defined as Household Food Security Survey Module (HFSSM) score ≥2)  **Homeless** in the past 12 months  Household monthly **income** before taxes per $100CAN increase | ≥95% cART adherence  High school and below: OR 0.98 (0.54, 1.78)  Currently working for pay: OR 0.91 (0.33, 2.49)  Food insecure: **OR 0.44 (0.24, 0.82)**  Homeless: OR 0.88 (0.43, 1.80)  Household monthly income: OR 1.05 (0.98, 1.13) | *Adjusted for age at interview date, household monthly income before taxes ($CAN), biological sex at birth, homeless in past 12 months, currently working for pay, Indigenous ancestry, highest level of education etc.*  ≥95% cART adherence  Food insecure: **aOR 0.47 (0.24, 0.93)** |
| Kyser et al 2011 [63] | 528 people living with HIV taking cART (USA) | **Education** (ref: high school or more)  **Employment** (ref: employed or retired) | cART non-adherence (“reporting having missed 1/more antiretroviral doses in past 3 days”)  <High school education: OR 1.86 (0.98, 3.54); p=0.057  Unemployed or looking for work: **OR 2.59 (1.53, 4.37); p<0.001** | *Adjusted for gender, race/ethnicity, duration since HIV diagnosis, current employment, health behaviours and mental health scores*  cART non-adherence  <high school education: aOR 1.39 (0.65, 2.95); p=0.393  Unemployed or looking for work: aOR 1.86 (0.99, 3.48); p=0.053  *Adjusted for race/ethnicity, duration since HIV diagnosis, alcohol use, aerobic exercise*  Unemployed or looking for work: **aOR 2.03 (1.14, 3.61); p=0.016** |
| Lacombe-Duncan et al 2019 [24] | 50 transwomen living with HIV (Canada) | **Education**  Insecure **housing** [defined as self-contained room, transition house, halfway house, safe house, couch surfing, outdoors on street, parks, or in a car. Ref: secure – apartment (own/rent) or a house (own/rent)]  <$20,000 personal **income** | ≥95% ART adherence  <High school education: Difference: -4.2 (-27.2, 26.3)  Insecure housing: Fisher's exact test 0.0 (-29.9, 22.6)  <$20,000: Fisher's exact test -14.0 (-43.7, 6.7) |  |
| McCoy et al 2016 [64] | 426 participants completed PRIME baseline study procedures and included in the analysis (USA) | **Educational** level [categorised as <high school graduate, HS graduate, some College, College graduate or higher)  **Employment** status [categorised as unemployed/disabled or working]  Annual **income** [categorised as >$20,000; $10,000-$20,000; $0-10,000] | ≥95% ART adherence  Educational level: OR 0.561 (0.301, 1.047); p=0.069  Employment status: OR 1.133 (0.621, 2.070); p=0.684  Annual income: **OR 1.267 (1.039, 1.545); p=0.019** |  |
| Mimiaga et al 2019 [29] | 296 adult residents of Rhode Island living with HIV (USA) | **Educational** attainment (ref: some College or more)  **Employment** (ref: employed)  **Homeless** in the past 12 months  No annual household **income** (ref: yes – categorised <$10,000; $10,000-19,999; $20,000-29,999; $30,000 or more)  **Financial** concerns in the past 12 months | Sub-optimal ART adherence – sometimes or never (vs. always adherent: 100%) take HIV medications as prescribed during past 12 months  <High school education: OR 2.62 (0.99, 7.00); p=0.06  High school degree or equivalent: OR 1.63 (0.54, 4.95); p=0.39  Not employed: OR 1.33 (0.57, 3.07); p=0.50  Homeless in past 12 months: OR 2.16 (0.80, 5.86); p=0.13  No income: OR 0.99 (0.33, 3.10); p=0.99  Financial concerns: OR 2.08 (0.93, 4.65); p=0.07 |  |
| Mohammed et al 2004 [65] | 273 people living with HIV from 8 areas in non-urban Louisiana (USA) | **Education** (ref: >high school)  **Employment** status | HAART non-adherence ("self-report of missing any doses of HAART medication in the prior week")  ≤High school: OR 0.63 (0.34, 1.14); p=0.12  Unemployed: OR 1.67 (0.86, 3.22); p=0.13 |  |
| Nyaku, Beer and Shu 2019 [66] | 18,423 people living with HIV who self-reported currently taking ART (USA including Puerto Rico) | **Education** (ref: >high school)  **Homeless** in the past 12 months  **Poverty** | ART non-persistence  ≤High school or equivalent: **OR 1.12 (1.00, 1.25); p=0.041^‡^**  Homeless in past 12 months: **OR 2.35 (2.02, 2.75); p=0.000^‡^**  Poverty: **OR 1.39 (1.24, 1.55); p=0.000^‡^** |  |
| O’Neil et al 2012 [67] | 566 people living with HIV who have accessed ART (Canada) | **Education** (ref: ≥high school)  Unstable **housing** [defined as living in a single room occupancy hotel, shelter, hostel, treatment centre, prison, or having no fixed address at the time of interview] (ref: stable housing - living in apartment/house)  Annual **income** ≤$15,000 (ref: >$15,000) | ≥95% HAART adherence  <High school: **OR 0.40 (0.29, 0.57); p=0.000^‡^**  Unstable housing: **OR 0.53 (0.37, 0.77); p=0.0006^‡^**  ≤$15,000 income: **OR 0.40 (0.28, 0.58); p=0.000^‡^** | *Adjusted for age, gender, injection drug use, enrolled in maximally assisted therapy programme*  ≥95% HAART adherence  <$15,000: **aOR 0.47 (0.31, 0.72); p<0.001** |
| Papadopoulou 2000 [68] | 56 participants taking cART (England, UK) | **Educational** background [categorised as left school at 15, vocational, A Levels, Diploma, Degree, MSc] | Adherence 1: “adhering to correct dose of each drug”  Educational background: χ² 1.431 (df =2); p=0.489  Adherence 2: “taking drugs at the right time”  Educational background: χ² 0.404 (df=2); p=0.817  Adherence 3: “following the dietary instructions”  Educational background: χ² 4.832 (df=2); p=0.089  Adherence 4: “overall adherence to taking the drugs as prescribed”  Educational background: χ² 1.233 (df=2); p=0.540 |  |
| Parruti et al 2006 [69] | 171 people living with HIV followed for at least 24 weeks (Italy) | **Occupation** (ref: employed – farmer/clerk/factory worker/dealer or consultant/freelance/entrepreneur)  **Homeless** familial status (ref: single/separated)  **Socioeconomic status** (ref: medium or high) | **≥**90% HAART adherence  Unemployed/retired: OR 0.89 (0.35, 2.26); p=0.802^‡^  Homeless: OR 2.25 (0.28, 18.0); p=0.444^‡^  Very low or low SES: OR 0.76 (0.30, 2.00); p=0.571^‡^ | *Adjusted for tolerance of therapy and >10 pills prescribed per day.*  <90% HAART adherence  Homeless: **aHR** **1.95 (1.02, 3.74); p=0.043** |
| Phillips 2011 [70] | 160 Black men living with HIV/AIDS who use illicit drugs (USA) | Unstable **housing** status, including homeless or marginally housed (ref: stably housed) | Self-reported ART adherence (“mean number of ART doses missed in last 4 days”)  Stably housed: **β (-0.86); p≤0.01** | *Adjusted for intermediary determinants (e.g. social cohesion, psychological distress, illicit drug use etc.)*  Self-reported ART adherence  Unstable housing: **β (-5.12) [95% CI -6.70, -4.32]; p≤0.01** |
| Phillips et al 2013 [71] | 1873 people living with HIV (Canada; US including Puerto Rico) | **Education** [categorised as 11th grade or less, high school, 2+ years College] | ≥99% ART adherence (self-reported visual analogue scale for 30 days)  Education: OR 0.968 (0.870, 1.077) |  |
| Saracino et al 2018 [37] | 8,023 people living with HIV (Italy) | **Education** (ref: elementary school)  **Employment** (ref: full-time worker) |  | *Adjusted for CD4 count, viral load, pregnancy status, smoking*  ART discontinuation  Junior high school: aHR 0.99 (0.84, 1.17); p=0.930  High school: aHR 1.01 (0.85, 1.19); p=0.923  University: aHR 1.08 (0.89, 1.32); p=0.420  Missing data (education): aHR 0.96 (0.81, 1.15); p=0.658  Unemployed: **aHR 1.18 (1.04, 1.34); p=0.008**  Self-employed: aHR 1.04 (0.94, 1.15); p=0.451  Temporary employed: aHR 1.00 (0.78, 1.28); p=0.985  Student: aHR 0.87 (0.69, 1.10); p=0.233  Retired: aHR 1.15 (0.94, 1.40); p=0.179  Housewife: **aHR 0.73 (0.59, 0.90); p=0.003**  Other/unknown (employment): aHR 1.20 (0.94, 1.52); p=0.143  Missing data (employment): aHR 0.93 (0.81, 1.070); p=0.313 |
| Sellier et al 2006 [72] | 61 people living with HIV reporting to 3 infectious diseases departments who were born in SSA but living in France (France) | **Education** (ref: secondary school or university)  **Employment** | ART adherence (never missing doses compared to frequently or rarely)  None or primary school education: **OR 0.234 (0.067, 0.818); p=0.023^‡^**  Unemployed: **OR 5.28 (1.51, 18.46); p=0.0092^‡^** |  |
| Sherr et al 2012 [73] | 259 adults living with HIV attending 5 HIV clinics (UK) | University level **education** | HAART adherence (all doses taken at correct time and under correct conditions)  University education (women): **OR 0.25 (0.09, 0.66)**  University education (men): OR 3.18 (1.02, 9.86) | *Adjusted for race/ethnicity, born in the UK, age (per unit increase), relational behaviour, symptoms, quality of life*  HAART adherence  University education (women): **aOR 0.26 (0.09, 0.76); p=0.014**  University education (men): aOR 2.64 (0.63, 10.94); p=0.182 |
| Siefried et al 2017 [74] | 522 participants at 7 sexual health, hospital, and general practice clinics (Australia) | **Employment**  Living in subsidised **housing**  **Income** <$580 per week  Required **financial** assistance in the previous 12 months  Going without for **financial** reasons (food, rent etc.)  **Cost** was a barrier to accessing healthcare | Sub-optimal ART adherence (“self-reported missed ≥3 ART in last 3 months”)  Unemployed: **OR 1.8 (1.1, 2.9); p=0.019**  Living in subsidised housing: OR 1.7 (1.0, 3.0); p=0.047  Income <$580 per week: **OR 2.0 (1.2, 3.2); p=0.007**  Required financial assistance: **OR 3.5 (2.1, 5.8); p<0.001**  Going without for financial reasons: **OR 3.8 (2.3, 6.3); p<0.001**  Cost was a barrier to accessing HIV healthcare: **OR 2.0 (1.1, 3.7); p=0.022** | *Adjusted for sociodemographic variables including not currently in a relationship and born in Australia*  Sub-optimal ART adherence  Living in subsidised housing: **aOR 2.5 (1.0, 6.2); p=0.045** |
| Singh et al 1999 [75] | 123 people as they completed a refill-methodology assessment of adherence (USA) | **Education** [categorised as grade school, technical school, high school, College, postgraduate]  **Employment**  **Income** ($ per month) [categorised as $0-500, $500-$1000, $1000-$1500, >$1500] | ART non-adherence (<90%)  Education: p=0.31  Unemployed: RR 1.30 (0.59, 2.8); p=0.52^‡‡^  Income: p=0.125 |  |
| Spire et al 2002 [76] | 445 people living with HIV who were enrolled in 47 French hospital departments delivering specialised care for HIV/AIDS patients (France) | **Housing** conditions (ref: stable)  Living on **welfare** | HAART non-adherence at 4 months follow up  Stable but poor housing at baseline: **OR 2.07 (1.26, 3.42)**  Unstable housing at baseline: **OR 3.92 (1.46, 3.73)**  Living on welfare: OR 1.80 (0.95, 3.41) | *Adjusted for M0 baseline variables including age, marital status or adjusted by M0/M4 evolution variables (e.g. tobacco/alcohol consumption etc.)*  HAART non-adherence at 4 months follow up (controlling for M0 variables)  Stable but poor housing at baseline: **aOR 1.88 (1.15, 3.08)**  Unstable housing at baseline: **aOR 2.76 (1.30, 5.85)**  HAART non-adherence at 4 months follow up (controlling for M0/M4 evolution variables)  Stable but poor housing at baseline: **aOR 1.71 (1.01, 2.92)**  Unstable housing at baseline: **aOR 2.70 (1.20, 6.08)** |
| Stone et al 2001 [77] | 289 women living with HIV/AIDS (USA) | **Education** (ref: 0-11 years) | HAART adherence (“missed ≥1 doses in past 3 days”)  12+ years education: OR 0.8 (0.5, 1.2) |  |
| Storholm et al 2019 [78] | 239 African American adults living with HIV recruited in community settings in Los Angeles (USA) | **Education**  **Employment** (full-time or part-time)  Currently stably **housed** [e.g. rent or own home/apartment, "publicly subsidised housing". Reference: unstable housing e.g. ("residential drug, alcohol or other treatment facility", “a friend or relative’s home or apartment,” “temporary or transitional housing,” or “homeless: sleeping in a shelter or on the street”)]  Annual **income** <$10,000 | ART adherence trajectory group: high-stable ART (mean >90%) compared to low-decreasing (mean <27%)  <High school education: OR 1.06 (0.45, 2.52)  Employed: OR 1.27 (0.36, 4.43)  Currently stably housed: OR 1.01 (0.48, 2.12)  <$10,000: OR 0.98 (0.49, 1.93)  ART adherence trajectory group: moderately low-stable (mean >60%) compared to low-decreasing (mean <27%)  <High school education: OR 0.88 (0.36, 2.19)  Employed: OR 1.45 (0.41, 5.09)  Currently stably housed: OR 0.93 (0.44, 1.99)  <$10,000: OR 1.21 (0.60, 2.47)  ART adherence trajectory group: high-stable (mean >90%) compared to moderately low-stable (mean >60%)  <High school education: OR 1.20 (0.54, 2.66)  Employed: OR 0.87 (0.31, 2.45)  Currently stably housed: OR 1.09 (0.55, 2.14)  <$10,000: OR 0.80 (0.43, 1.51) |  |
| Sunil and McGehee 2007 [79] | 1910 participants taking ART (USA) | **Education** (ref: some high school, no degree) |  | *Stratified by race/ethnicity and adjusted for by gender, age, measures of religion, social support*  ART adherence (adherent: “took all medications exactly as prescribed in the past week; non-adherent: if missed one medication in the past week”)  High school (White): **aOR 1.30 (1.239, 1.365); p<0.001**  Some College (White): **aOR 1.41 (1.344, 1.481); p<0.001**  BA, BS (White): **aOR 1.58 (1.505, 1.661); p<0.001**  High school (African American): **aOR 1.96 (1.873, 2.054); p<0.001**  Some College (African American): **aOR 1.45 (1.383, 1.524); p<0.001**  BA, BS (African American): **aOR 0.88 (0.819, 0.952); p<0.005**  High school (Hispanic): **aOR 0.78 (0.72, 0.84); p<0.001**  Some College (Hispanic): **aOR 0.71 (0.661, 0.772); p<0.001**  BA, BS (Hispanic): **aOR 1.40 (1.268, 1.553); p<0.001** |
| Surratt et al 2015 [80] | 503 socioeconomically disadvantaged substance users living with HIV in urban South Florida (USA) | **Homeless**, past 90 days  Current **housing** type (ref: own/rent house or apartment)  **Poverty** level (mean)  **Neighbourhood disorder** (mean)  Monthly **income** <$1000 (ref: ≥$1000) | ARV non-adherence (missed ARVs because of diversion in past 90 days)  Homeless: **OR 1.97 (1.34, 2.90); p≤0.001**  Public housing: OR 0.94 (0.54, 1.64)  Residential facility: OR 0.44 (0.15, 1.32)  Staying with friend or relative: **OR 2.41 (1.33, 4.39); p≤0.001**  Boarding house or hotel: OR 1.82 (0.51, 6,52)  Shelter: **OR 2.48 (1.46, 4.21); p≤0.001**  Street location: OR 9.56 (0.97, 94.29)  Poverty level: **OR 0.98 (0.96, 0.99); p≤0.05**  Neighbourhood disorder: **OR 1.03 (1.01, 1.05); p≤0.01**  <$1000: OR 0.96 (0.59, 1.56) | *Adjusted for age, gender, race/ethnicity, income, and substance dependence (and 1 mediator - neighbourhood disorder, recent homelessness)*  ARV non-adherence  Homeless: **aOR** **1.74 (1.15, 2.63); p=0.008**  Neighbourhood disorder: aOR 1.02 (1.00, 1.04); p=0.098 |
| Uusküla et al 2012 [81] | 144 people living with HIV presenting for routine HIV clinical care visits (Estonia) | **Education** (ref: ≤ 9 years)  Main source of **income** (ref: full-/part-time work) | ART non-adherence (<100%)  >9 years education: OR 2.31 (0.60, 13.17); p=0.273  Income (social benefits): OR 0.55 (0.14, 2.16); p=0.356  Income (relatives/partners): OR 1.16 (0.25, 5.18); p>0.9 | *Adjusted for health insurance status and self-rated health.*  ART non-adherence (<100%)  >9 years education: aOR 2.76 (0.81, 12.83); p=0.137  Income (social benefits): aOR 0.50 (0.14, 1.91); p=0.291  Income (relatives/partners): aOR 1.40 (0.32, 6.13); p=0.649 |
| Vyas et al 2014 [82] | 350 adults living with HIV attending an HIV clinic in San Diego (USA) | Annual household **income** ≥$10,000USD |  | *Adjusted for religious practices and beliefs*  ≥90% ART adherence  ≥$10,000: **aOR** **2.37 (1.10, 5.14); p=0.004** |
| Weiser et al 2013 [45] | 284 homeless and marginally housed individuals living with HIV (USA) | **Education**  Any **food** **insecurity** [including mild, moderate, or severe food insecurity (ref: food secure)]  Recent **homelessness** [defined as living in shelter or on street in past 90 days]  **Income** <median ($916) | <90% ART adherence  <High school education: OR 1.05 (0.73, 1.52)  Any food insecurity: **OR 1.60 (1.29, 1.99); p<0.001**  Recent homelessness: **OR 1.75 (1.16, 2.64); p<0.001**  Income <median: OR 1.21 (0.86, 1.72) | *Adjusted for age, sex, ethnicity and other intermediary variables*  <90% ART adherence  Any food insecurity: **aOR 1.48 (1.19, 1.85); p<0.001**  Recent homelessness: **aOR** **1.55 (1.04, 2.32); p<0.05** |
| Yang and Bang 2017 [83] | 300 people living with HIV from six hospitals (South Korea) | **Education** (ref: College and above)  **Employment** [categorised as no job, full-time, temporary worker, daily worker, business owner]  Monthly **income** ($USD) [categorised as <$500, $500-1000, $1000-1500, $1500-2000, ≥$2000] | ≥95% ART adherence  Elementary school: OR 2.42 (0.69, 8.48); p=0.17  Middle school: OR 0.92 (0.24, 3.47); p=0.90  High school: OR 0.58 (0.29, 1.17); p=0.13  Education: **χ² (10.773); p=0.013**  Occupation: χ² (2.908); p=0.573  Monthly income: χ² (7.496); p=0.112 |  |

Abbreviations: AFDC: Aid to Families with Dependent Children; CAN: Canadian dollar; EPICES: Evaluation of Precariousness and Inequalities in Health Examination Centres; GA: General Assistance; GED: General Educational Diploma; HFSSM: Household Food Security Module; MED-OUT: pharmacy-refill based index measure referring to days out of medication; SRO: Single Room Occupancy; SSI/SSDI: Social Security, Supplemental Security Income/Social Security Disability Insurance; USD: United States dollar

**References**

1. Almeida-Brasil CC, Moodie EEM, McLinden T, Hamelin A-M, Walmsley SL, Rourke SB, et al. Medication nonadherence, multitablet regimens, and food insecurity are key experiences in the pathway to incomplete HIV suppression. AIDS [Internet]. 2018;32(10). Available from: https://journals.lww.com/aidsonline/Fulltext/2018/06190/Medication_nonadherence,_multitablet_regimens,_and.13.aspx

2. Anderson JC, Campbell JC, Glass NE, Decker MR, Perrin N, Farley J. Impact of intimate partner violence on clinic attendance, viral suppression and CD4 cell count of women living with HIV in an urban clinic setting. AIDS Care. 2018;30(4):399–408.

3. Baguso GN, Turner CM, Santos G-M, Raymond HF, Dawson-Rose C, Lin J, et al. Successes and final challenges along the HIV care continuum with transwomen in San Francisco. Journal of the International AIDS Society. 2019 Apr 1;22(4):e25270.

4. Blank AE, Fletcher J, Verdecias N, Garcia I, Blackstock O, Cunningham C. Factors associated with retention and viral suppression among a cohort of HIV+ women of color. AIDS Patient Care STDS. 2015;29 Suppl 1(Suppl 1):S27–35.

5. Burch LS. Impact of gender, sexual orientation and socio-economic factors on HIV treatment outcomes in the UK [Internet] [Thesis (Doctoral)]. University of London, University College London (United Kingdom); 2018. Available from: https://discovery.ucl.ac.uk/id/eprint/10041026

6. Clemenzi-Allen A, Geng E, Christopoulos K, Hammer H, Buchbinder S, Havlir D, et al. Degree of Housing Instability Shows Independent ‘Dose-Response’ With Virologic Suppression Rates Among People Living With Human Immunodeficiency Virus. Open Forum Infect Dis. 2018;5(3):ofy035–ofy035.

7. Collazos J, Asensi V, Carton JA, Ibarra S. The influence of the patients’ educational levels on socioeconomic, clinical, immunological and virological endpoints. AIDS Care. 2009;21(4):511–9.

8. D’Almeida KW, Lert F, Spire B, Dray-Spira R. Determinants of virological response to antiretroviral therapy: socio-economic status still plays a role in the era of cART. Results from the ANRS-VESPA 2 study, France. Antiviral therapy. 2016;21(8):661–70.

9. Del Amo on behalf of The Socio-economic Inequalities and HIV Working Group for Collaboration of Observational HIV Epidemiological Research in Europe (COHERE) in Euro-Coord. Inequalities by educational level in response to combination antiretroviral treatment and survival in HIV-positive men and women in Europe. AIDS. 2017;31(2):253–62.

10. Doshi RK, Milberg J, Jumento T, Matthews T, Dempsey A, Cheever LW. For Many Served By The Ryan White HIV/AIDS Program, Disparities In Viral Suppression Decreased, 2010-14. Health Affairs. 2017;36(1):116–23.

11. Dowshen N, Matone M, Luan X, Lee S, Belzer M, Fernandez MI, et al. Behavioral and Health Outcomes for HIV+ Young Transgender Women (YTW) Linked To and Engaged in Medical Care. LGBT health. 2016;3(2):162–7.

12. Fadul N, Taylor P, Kearney G. Predictors of viral load suppression in HIV-infected patient in rural Eastern North Carolina. Open Forum Infectious Diseases. 2017;4(Supplement 1):S421.

13. Feldman M, Thomas J, Alexy E, Irvine M. Crystal methamphetamine use and HIV medical outcomes among HIV-infected men who have sex with men accessing support services in New York. Drug and Alcohol Dependence. 2015;147:266–71.

14. Feller DJ, Agins BD. Understanding Determinants of Racial and Ethnic Disparities in Viral Load Suppression: A Data Mining Approach. J Int Assoc Provid AIDS Care. 2016 Sep 14;16(1):23–9.

15. Gardner S., Rachlis B., Light L., Raboud J., Cooper C., Kendall C., et al. Impact of social determinants of health and substance use on HIV viral suppression (SVL). Canadian Journal of Infectious Diseases and Medical Microbiology. 2015;26(SUPPL. SB):38B.

16. Gueler A, Schoeni-Affolter F, Moser A, Bertisch B, Bucher HC, Calmy A, et al. Neighbourhood socio-economic position, late presentation and outcomes in people living with HIV in Switzerland. AIDS (London, England). 2015;29(2):231–8.

17. Haider MR, Brown MJ, Harrison S, Yang X, Ingram L, Bhochhibhoya A, et al. Sociodemographic factors affecting viral load suppression among people living with HIV in South Carolina. AIDS Care. 2019;1–9.

18. Hussen SA, Easley KA, Smith JC, Shenvi N, Harper GW, Camacho-Gonzalez AF, et al. Social Capital, Depressive Symptoms, and HIV Viral Suppression Among Young Black, Gay, Bisexual and Other Men Who Have Sex with Men Living with HIV. AIDS and Behavior. 2018;22(9):3024–32.

19. Iralu J, Duran B, Pearson CR, Jiang YZ, Foley K, Harrison M. Risk factors for HIV disease progression in a rural southwest American Indian population. Public Health Reports. 2010;125(Supplement 4):43–50.

20. Jansen K., Brockmeyer N.H., Dlugay V., Stoll M., Pauli R., Golz J., et al. Poorer clinical outcomes of HIV-positive patients (PLWHA) having lower income: Results of an analysis on the basis of the KompNet cohort. HIV Medicine. 2009;10(SUPPL. 2):153.

21. Kacanek D, Huo YanLing, Malee K, Mellins CA, Smith R, Garvie PA, et al. Nonadherence and unsuppressed viral load across adolescence among US youth with perinatally acquired HIV. AIDS. 2019;33(12):1923–34.

22. Kalichman S, Hernandez D, Cherry C, Kalichman M, Washington C, Grebler T. Food Insecurity and Other Poverty Indicators Among People living with HIV/AIDS: Effects on Treatment and Health Outcomes. Journal of Community Health. 2014;39(6):1133–9.

23. Kalichman SC, Cherry C, Amaral C, White D, Kalichman MO, Pope H, et al. Health and treatment implications of food insufficiency among people living with HIV/AIDS, Atlanta, Georgia. J Urban Health. 2010;87(4):631–41.

24. Lacombe-Duncan A., Bauer G.R., Logie C.H., Newman P.A., Shokoohi M., Kay E.S., et al. The HIV Care Cascade among Transgender Women with HIV in Canada: A Mixed-Methods Study. AIDS Patient Care and STDs. 2019;33(7):308–22.

25. Lim S, Nash D, Hollod L, Harris TG, Lennon MC, Thorpe LE. Influence of Jail Incarceration and Homelessness Patterns on Engagement in HIV Care and HIV Viral Suppression among New York City Adults Living with HIV/AIDS. PLOS ONE. 2015;10(11):e0141912.

26. Ludema C, Cole SR, Eron JJJ, Edmonds A, Holmes GM, Anastos K, et al. Impact of Health Insurance, ADAP, and Income on HIV Viral Suppression Among US Women in the Women’s Interagency HIV Study, 2006-2009. JAIDS. 2016;73(3):307–12.

27. Marshall BDL, Elston B, Dobrer S, Parashar S, Hogg RS, Montaner JSG, et al. The population impact of eliminating homelessness on HIV viral suppression among people who use drugs. AIDS. 2016;30(6):933–41.

28. Miller CL, Spittal PM, Wood E, Chan K, Schechter MT, Montaner JSG, et al. Inadequacies in antiretroviral therapy use among Aboriginal and other Canadian populations. AIDS Care. 2006;18(8):968–76.

29. Mimiaga MJ, August Oddleifson D, Meersman SC, Silvia A, Hughto JMW, Landers S, et al. Multilevel Barriers to Engagement in the HIV Care Continuum Among Residents of the State of Rhode Island Living with HIV. AIDS and behavior. 2019;(9712133).

30. Moore DM, Cui Z, Lachowsky N, Raymond HF, Roth E, Rich A, et al. HIV Community Viral Load and Factors Associated With Elevated Viremia Among a Community-Based Sample of Men Who Have Sex With Men in Vancouver, Canada. JAIDS. 2016;72(1):87–95.

31. Oliver C, Rebeiro PF, Hopkins MJ, Byram B, Carpenter L, Clouse K, et al. Substance Use, Demographic and Socioeconomic Factors Are Independently Associated With Postpartum HIV Care Engagement in the Southern United States, 1999-2016. Open forum infectious diseases. 2019;6(2):ofz023.

32. Persson L, Gullberg B, Hanson BS, Moestrup T, Ostergren PO. HIV infection: social network, social support, and CD4 lymphocyte values in infected homosexual men in Malmo, Sweden. Journal of epidemiology and community health. 1994;48(6):580–5.

33. Raho-Moussa M, Guiguet M, Michaud C, Honoré P, Palacios C, Boué F, et al. Respective roles of migration and social deprivation for virological non-suppression in HIV-infected adults on antiretroviral therapy in France. PLOS ONE. 2019 Mar 7;14(3):e0213019.

34. Rebeiro PF, Howe CJ, Rogers WB, Bebawy SS, Turner M, Kheshti A, et al. The relationship between adverse neighborhood socioeconomic context and HIV continuum of care outcomes in a diverse HIV clinic cohort in the Southern United States. AIDS care. 2018;30(11):1426–34.

35. Robinson AC, Knowlton AR. Gender Differences in Psychosocial Factors Associated with HIV Viral Suppression Among African-American Injection Drug Users. AIDS Behav. 2016 Feb;20(2):385–94.

36. Santos GM, Wilson EC, Rapues J, Macias O, Packer T, Raymond HF. HIV treatment cascade among transgender women in a San Francisco respondent driven sampling study. Sexually Transmitted Infections. 2014;90(5):430–3.

37. Saracino A, Zaccarelli M, Lorenzini P, Bandera A, Marchetti G, Castelli F, et al. Impact of social determinants on antiretroviral therapy access and outcomes entering the era of universal treatment for people living with HIV in Italy. BMC public health. 2018;18(1):870.

38. Sayles JN, Rurangirwa J, Kim M, Kinsler J, Oruga R, Janson M. Operationalizing Treatment as Prevention in Los Angeles County: Antiretroviral Therapy Use and Factors Associated with Unsuppressed Viral Load in the Ryan White System of Care. AIDS Patient Care & STDs. 2012;26(8):463–70.

39. Schafer KR, Brant J, Gupta S, Thorpe J, Winstead-Derlega C, Pinkerton R, et al. Intimate Partner Violence: A Predictor of Worse HIV Outcomes and Engagement in Care. AIDS Patient Care and STDs. 2012;26(6):356–65.

40. Shacham E., Nurutdinova D., Onen N., Stamm K., Overton E.T. The interplay of sociodemographic factors on virologic suppression among a U.S. outpatient HIV clinic population. AIDS Patient Care and STDs. 2010;24(4):229–35.

41. Shacham E, Lian M, Onen NF, Donovan M, Overton ET. Are neighborhood conditions associated with HIV management?. HIV medicine. 2013;14(10):624–32.

42. Sobrino-Vegas P, Rodriguez-Urrego J, Berenguer J, Caro-Murillo AM, Blanco JR, Viciana P, et al. Educational gradient in HIV diagnosis delay, mortality, antiretroviral treatment initiation and response in a country with universal health care. Antiviral therapy. 2012;17(1):1–8.

43. Tymejczyk O, Jamison K, Pathela P, Braunstein S, Schillinger JA, Nash D. HIV care and viral load suppression after sexual health clinic visits by out-of-care HIV-positive persons. AIDS Patient Care and STDs. 2018;32(10):390–8.

44. Wagoner N, Elopre L, Westfall A, Mugavero M, Turan J, Hook E. Reported Church Attendance at the Time of Entry into HIV Care is Associated with Viral Load Suppression at 12 Months. AIDS & Behavior. 2016;20(8):1706–12.

45. Weiser SD, Yuan C, Guzman D, Frongillo EA, Riley ED, Bangsberg DR, et al. Food insecurity and HIV clinical outcomes in a longitudinal study of urban homeless and marginally housed HIV-infected individuals. AIDS. 2013;27(18):2953–8.

46. Whelan BM, Hebert PL, Ahrens KR, Katz DA, Buskin SE, Golden MR, et al. Predictors of failure to reach viral suppression within 1 year after human immunodeficiency virus diagnosis: a surveillance-based analysis. Sexually Transmitted Diseases. 2019;46(11):728–32.

47. Wilson EC, Turner C, Arayasirikul S, Woods T, Nguyen T, Lin R, et al. Housing and income effects on HIV-related health outcomes in the San Francisco Bay Area - findings from the SPNS transwomen of color initiative. AIDS Care. 2018;30(11):1356–9.

48. Yehia B.R., Schranz A.J., Momplaisir F., Keller S.C., Gross R., Frank I., et al. Outcomes of HIV-infected patients receiving care at multiple clinics. AIDS and behavior. 2014;18(8):1511–22.

49. Abgrall S, Fugon L, Lele N, Carde E, Bentata, Patey O, et al. Risk factors for adherence failure in HIV-infected sub-Saharan migrants living in France and travelling back to their native country. International Journal of STD and AIDS. 2014;25(5):389–90.

50. Berg KM, Demas PA, Howard AA, Schoenbaum EE, Gourevitch MN, Arnsten JH. Gender differences in factors associated with adherence to antiretroviral therapy. J Gen Intern Med. 2004;19(11):1111–7.

51. Carrieri M, Chesney M, Spire B, Loundou A, Sobel, Lepeu G., et al. Failure to maintain adherence to HAART in a cohort of French HIV-positive injecting drug users. International Journal of Behavioral Medicine. 2003;10(1):1–14.

52. Chitsaz E., Meyer J.P., Krishnan A., Springer S.A., Marcus R., Zaller N., et al. Contribution of substance use disorders on HIV treatment outcomes and antiretroviral medication adherence among HIV-infected persons entering jail. AIDS and behavior. 2013;17(Supplement 2):S118–27.

53. Creasy SL, Henderson ER, Bukowski LA, Matthews DD, Stall RD, Hawk ME. HIV Testing and ART Adherence Among Unstably Housed Black Men Who Have Sex with Men in the United States. AIDS & Behavior. 2019;23(11):3044–51.

54. Dorz S, Lazzarini L, Cattelan A, Meneghetti F, Novara C, Concia E, et al. Evaluation of adherence to antiretroviral therapy in Italian HIV patients. AIDS Patient Care & STDs. 2003;17(1):33–41.

55. Gebo KA, Keruly J, Moore RD. Association of social stress, illicit drug use, and health beliefs with nonadherence to antiretroviral therapy. Journal of General Internal Medicine. 2003;18(2):104–11.

56. Golin CE, Liu H, Hays RD, Miller LG, Beck CK, Ickovics J, et al. A prospective study of predictors of adherence to combination antiretroviral medication. J Gen Intern Med. 2002;17(10):756–65.

57. Gordillo V, del Amo J, Soriano V, Gonzalez-Lahoz J. Sociodemographic and psychological variables influencing adherence to antiretroviral therapy. AIDS. 1999;13(13):1763–9.

58. Johnson M, Catz S, Remien RH, Rotheram-Borus M, Morin, Charlebois E, et al. Theory-guided, empirically supported avenues for intervention on HIV medication nonadherence: findings from the Healthy Living Project. AIDS Patient Care & STDs. 2003;17(12):645–56.

59. Kalichman SC, Grebler T. Stress and poverty predictors of treatment adherence among people with low-literacy living with HIV/AIDS. Psychosomatic medicine. 2010;72(8):810–6.

60. Keith McInnes D, Shimada SL, Rao SR, Quill A, Duggal M, Gifford AL, et al. Personal health record use and its association with antiretroviral adherence: survey and medical record data from 1871 US veterans infected with HIV. AIDS and behavior. 2013;17(9):3091–100.

61. Kleeberger CA, Buechner J, Palella F, Detels R, Riddler S, Godfrey R, et al. Changes in adherence to highly active antiretroviral therapy medications in the Multicenter AIDS Cohort Study*. AIDS [Internet]. 2004;18(4). Available from: https://journals.lww.com/aidsonline/Fulltext/2004/03050/Changes_in_adherence_to_highly_active.13.aspx

62. Koehn K, McLinden T, Collins AB, McDougall P, Baltzer-Turje R, Miewald C, et al. Assessing the impact of food insecurity on HIV medication adherence in the context of an integrated care programme for people living with HIV in Vancouver, Canada. Public Health Nutrition. 2020 Mar;23(4):683–90.

63. Kyser M, Buchacz K, Bush TJ, Conley LJ, Hammer J, Henry K, et al. Factors associated with non-adherence to antiretroviral therapy in the SUN study. AIDS Care. 2011;23(5):601–11.

64. McCoy K, Waldrop-Valverde D, Balderson BH, Mahoney C, Catz S. Correlates of Antiretroviral Therapy Adherence among HIV-Infected Older Adults. Journal of the International Association of Providers of AIDS Care. 2016;15(3):248–55.

65. Mohammed H, Kieltyka L, Richardson-Alston G, Magnus M, Fawal H, Vermund SH, et al. Adherence to HAART Among HIV-Infected Persons in Rural Louisiana. AIDS Patient Care and STDs. 2004;18(5):289–96.

66. Nyaku M, Beer L, Shu F. Non-persistence to antiretroviral therapy among adults receiving HIV medical care in the United States. AIDS Care. 2019;31(5):599–608.

67. O’Neil CR, Palmer AK, Coulter S, O’Brien N, Shen A, Zhang W, et al. Factors associated with antiretroviral medication adherence among HIV-positive adults accessing highly active antiretroviral therapy (HAART) in British Columbia, Canada. Journal of the International Association of Physicians in AIDS Care. 2012;11(2):134–41.

68. Papadopoulou A. Adherence to combination therapies in people with HIV/AIDS [Internet]. [Ann Arbor]: University of London, University College London (United Kingdom); 2000. Available from: http://iclibezp1.cc.ic.ac.uk/login?url=https://search.proquest.com/docview/1759198035?accountid=16260

69. Parruti G., Manzoli L., Marani Toro P., D’Amico G., Rotolo S., Graziani V., et al. Long-term adherence to first-line highly active antiretroviral therapy in a hospital-based cohort: Predictors and impact on virologic response and relapse. AIDS Patient Care and STDs. 2006;20(1):48–57.

70. Phillips JC. Antiretroviral Therapy Adherence: Testing a Social Context Model Among Black Men Who Use Illicit Drugs. Journal of the Association of Nurses in AIDS Care. 2011;22(2):100–27.

71. Phillips JC, Webel A, Rose CD, Corless IB, Sullivan KM, Voss J, et al. Associations between the legal context of HIV, perceived social capital, and HIV antiretroviral adherence in North America. BMC Public Health. 2013;13(1):1–16.

72. Sellier P, Clevenbergh P, Ljubicic L, Simoneau G, Evans J, Delcey V, et al. Comparative evaluation of adherence to antiretroviral therapy in sub-Saharan African native HIV-infected patients in France and Africa. Clinical infectious diseases. 2006;43(5):654–7.

73. Sherr L, Clucas C, Lampe F, Harding R, Johnson M, Fisher M, et al. Gender and Mental Health Aspects of Living with HIV Disease and Its Longer-Term Outcomes for UK Heterosexual Patients. Women & Health. 2012;52(3):214–33.

74. Siefried KJ, Mao L, Kerr S, Cysique LA, Gates TM, McAllister J, et al. Socioeconomic factors explain suboptimal adherence to antiretroviral therapy among HIV-infected Australian adults with viral suppression. PLOS ONE. 2017 Apr 3;12(4):e0174613.

75. Singh N, Berman SM, Swindells S, Justis JC, Mohr JA, Squier C, et al. Adherence of Human Immunodeficiency Virus—Infected Patients to Antiretroviral Therapy. Clinical Infectious Diseases. 1999;29(4):824–30.

76. Spire B, Duran S, Souville M, Leport C, Raffi F, Moatti J-P, et al. Adherence to highly active antiretroviral therapies (HAART) in HIV-infected patients: from a predictive to a dynamic approach. Social science & medicine. 2002;54(10):1481–96.

77. Stone VE, Hogan JW, Schuman P, Rompalo AM, Howard AA, Korkontzelou C, et al. Antiretroviral regimen complexity, self-reported adherence, and HIV patients’ understanding of their regimens: survey of women in the her study. JAIDS. 2001;28(2):124–31.

78. Storholm ED, Bogart LM, Mutchler MG, Klein DJ, Ghosh-Dastidar B, McDavitt B, et al. Antiretroviral Adherence Trajectories Among Black Americans Living with HIV. AIDS and behavior. 2019;23(8):1985–97.

79. Sunil TS, McGehee MA. Social and religious support on treatment adherence among HIV/AIDS patients by race/ethnicity. Journal of HIV/AIDS & Social Services. 2007;6(1/2):83–99.

80. Surratt HL, Kurtz SP, Levi-Minzi MA, Chen MX. Environmental influences on HIV medication adherence: the role of neighborhood disorder. American Journal of Public Health. 2015;105(8):1660–6.

81. Uuskula A, Laisaar K-T, Raag M, Smidt J, Semjonova S, Kogan J, et al. Antiretroviral therapy (ART) adherence and correlates to nonadherence among people on ART in Estonia. AIDS care. 2012;24(12):1470–9.

82. Vyas KJ, Limneos J, Qin HF, Mathews WC. Assessing baseline religious practices and beliefs to predict adherence to highly active antiretroviral therapy among HIV-infected persons. AIDS Care. 2014;26(8):983–7.

83. Yang HJ, Bang JH. Factors associated with medication adherence in patients with human immunodeficiency virus in South Korea. AIDS Care. 2017;29(10):1315–9.

1. Bold indicates statistical significance [↑](#endnote-ref-1)
2. Calculated by manuscript authors using data taken from article [↑](#endnote-ref-2)
3. Also report relative risks of someone living in stable housing and <47 and >47 years old in relation to intermediary determinants (insurance status, substance use) [↑](#endnote-ref-3)
4. Also report relative risks of someone without stable housing in relation to substance use [↑](#endnote-ref-4)
5. Also report relative risk of someone with unknown housing status in relation to substance use [↑](#endnote-ref-5)
6. Manuscript authors corrected upper 95% CI from that reported in original paper [↑](#endnote-ref-6)
